# Global profiling of alternative RNA splicing events provides insights into molecular differences between various types of hepatocellular carcinoma

**DOI:** 10.1186/s12864-016-3029-z

**Published:** 2016-08-26

**Authors:** Marie-Pier Tremblay, Victoria E. S. Armero, Andréa Allaire, Simon Boudreault, Camille Martenon-Brodeur, Mathieu Durand, Elvy Lapointe, Philippe Thibault, Maude Tremblay-Létourneau, Jean-Pierre Perreault, Michelle S. Scott, Martin Bisaillon

**Affiliations:** 1 Département de biochimie, Pavillon de recherche appliquée sur le cancer, Faculté de médecine et des sciences de la santé, Université de Sherbrooke, 3201 Jean-Mignault, Sherbrooke, QC J1E 4K8 Canada; 2 Plateforme RNomique, Université de Sherbrooke, Sherbrooke, QC J1E 4K8 Canada

**Keywords:** Liver cancer, Hepatitis B virus, Hepatitis C virus, RNA splicing, Gene dysregulation

## Abstract

**Background:**

Dysregulations in alternative splicing (AS) patterns have been associated with many human diseases including cancer. In the present study, alterations to the global RNA splicing landscape of cellular genes were investigated in a large-scale screen from 377 liver tissue samples using high-throughput RNA sequencing data.

**Results:**

Our study identifies modifications in the AS patterns of transcripts encoded by more than 2500 genes such as tumor suppressor genes, transcription factors, and kinases. These findings provide insights into the molecular differences between various types of hepatocellular carcinoma (HCC). Our analysis allowed the identification of 761 unique transcripts for which AS is misregulated in HBV-associated HCC, while 68 are unique to HCV-associated HCC, 54 to HBV&HCV-associated HCC, and 299 to virus-free HCC. Moreover, we demonstrate that the expression pattern of the RNA splicing factor hnRNPC in HCC tissues significantly correlates with patient survival. We also show that the expression of the HBx protein from HBV leads to modifications in the AS profiles of cellular genes. Finally, using RNA interference and a reverse transcription-PCR screening platform, we examined the implications of cellular proteins involved in the splicing of transcripts involved in apoptosis and demonstrate the potential contribution of these proteins in AS control.

**Conclusions:**

This study provides the first comprehensive portrait of global changes in the RNA splicing signatures that occur in hepatocellular carcinoma. Moreover, these data allowed us to identify unique signatures of genes for which AS is misregulated in the different types of HCC.

**Electronic supplementary material:**

The online version of this article (doi:10.1186/s12864-016-3029-z) contains supplementary material, which is available to authorized users.

## Background

Hepatocellular carcinoma (HCC) represents the fifth most prevalent tumor type and the third leading cause of cancer-related deaths around the world [1]. Chronic infection with hepatitis B virus (HBV) and hepatitis C virus (HCV) are the major causes of HCC worldwide [2]. These two hepatotropic viruses represent the most important risk factors for the development of HCC, with an estimated 80 % of all HCC cases globally [3].

HBV and HCV induce carcinogenesis via different molecular mechanisms. HBV infection causes HCC via both indirect and direct pathways [4]. HBV infection promotes chronic injury to liver cells, with constant necro-inflammation and regeneration activity [5]. As a consequence, an increase in hepatocyte turnover is observed which leads to the accumulation of potential critical mutations with subsequent malignant transformation and clonal expansion, leading to HCC [6]. Moreover, HBV infection can directly lead to HCC by integrating the viral genomic DNA into the host genome [7]. HBV integration can lead to numerous mutagenic consequences, including deletions, translocation, large inverted duplications, and amplifications, resulting in chromosomal instability [8]. In contrast, HCV does not integrate into the hepatocyte genome. The central hypothesis for HCV carcinogenesis is that it occurs via indirect pathways through chronic inflammation and hepatocellular injury. This is supported by the fact that cirrhosis is frequently a pre-requisite for HCV-induced HCC [4]. However, HCC has been reported to develop in a very small proportion of non-cirrhotic patients, suggesting a more direct effect from HCV itself [9]. Accordingly, various HCV proteins have been shown to have a role in the development of HCC development in various experimental models [10].

Alterations in RNA splicing of a small number of cellular genes have been observed in HCC, and although still limited, the available data suggest that splicing defects may play a role in hepatocarcinogenesis [11]. Higher eukaryotes use alternative splicing (AS) to change the composition of transcripts encoded by a gene through selection of different exons to be included in the mature mRNA, thus producing a variability at the proteomic level [12]. AS therefore diversifies the cellular proteome because it results in multiple transcripts and, as a consequence, various proteins are created from a single gene. These different proteins produced from the same gene can frequently promote different or even opposite biological effects. As a result, the relative abundance of each isoform may have consequences on cellular functions. An example of this is clearly demonstrated by the BCL-x gene. AS of the Bcl-x transcript results in two different mature mRNAs: Bcl-x(L), which has anti-apoptotic effects, and Bcl-x(S) which promotes apoptosis [13]. Therefore, it is not surprising that AS is tightly regulated and that variations in splicing patterns have been associated with many human diseases including cancer [14]. During the last few years, cancer-specific splice variants and cancer-associated changes in the relative levels of spliced isoforms of genes with a recognized role in carcinogenesis have been observed [15]. AS alterations can confer selective advantages to tumors, such as angiogenesis, proliferation, cell invasion and avoidance of apoptosis [16]. Recent evidences indicate that some of these can be used as prognostic or diagnostic biomarkers, and the development of strategies to correct and/or inhibit pathological splicing events will be key in order to develop future therapeutic approaches [17].

Various studies have shown alterations in the AS patterns of a limited number of cellular genes in HCC [11]. One interesting case of dysregulated AS occurring in HCC is the overexpression of a splice variant of DNA methyltransferase 3b (DNMT3b), namely DNMT3b4 [18]. An elevation of the ratio of DNMT3b4 to DNMT3b3 mRNA is correlated with DNA hypomethylation on pericentromeric satellite regions which may induce chromosomal instability and is considered an early event during hepatocarcinogenesis [18]. Other examples of aberrantly-spliced genes detected in HCC include the serine/threonine kinase aurora kinase B (AURKB), the E3 ubiquitin ligase, the p53-antagonistic protein MDM2, the cell surface adhesion cadherin 17 protein, and the Hugl-1, Klf6, and p73 tumor suppressors [11]. These examples illustrate the oncogenic potential of aberrantly spliced isoforms in HCC tissues.

In the present study, alterations to the global cellular AS landscape of more than 377 liver tissue samples from The Cancer Genome Atlas (TCGA) were investigated using high-throughput RNA sequencing data. This study provides a comprehensive portrait of global changes in the RNA splicing signatures that occur in HCC. We identify modifications in the AS patterns of transcripts encoded by more than 2500 genes such as transcription factors, tumor suppressor genes, kinases, and splicing factors. These findings allowed the identification of unique gene signatures for which AS is misregulated in different types of HCC.

## Results

### Modification of the cellular AS landscape in HCC

**Fig. 1:**
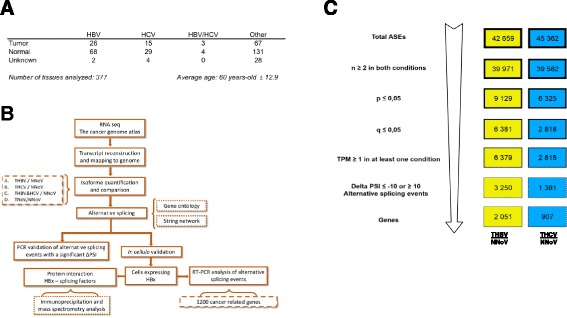
Alternative splicing of HBV- and HCV-associated hepatocellular carcinoma. **a** Classification of the TCGA RNA sequencing data for HCC and healthy tissues. **b** Overview of the strategy used to identify the changes in splicing for each type of HCC. **c** The splicing events list for HBV-associated HCC (THBV/NNoV) and HCV-associated HCC (THCV/NNoV) was filtered to keep only significant ASEs

Alterations to the global RNA splicing landscape of more than 377 liver tissue samples (Fig. 1a) from The Cancer Genome Atlas (TCGA) were investigated using high-throughput RNA sequencing data. The overview of all the analyses performed in this study is outlined in Fig. 1b. RNA-seq data were collected and mapped to the reference genome, followed by transcript assembly and analysis of RNA isoform abundance. We initially focused our study on HBV-associated HCC (THBV, Tumors with HBV) and HCV-associated HCC (THCV, Tumors with HCV), and we compared the global splicing patterns with normal tissues (NNoV, Normal, no virus). In order to identify the cellular AS patterns that are altered in HCC, we evaluated the modifications in splice abundances by quantifying all the primary transcripts that generate two or more isoforms. Alternative splicing events (ASEs) were detected and quantified using the percent-spliced-in (PSI) metric based on isoform expression (transcript-per-million, TPM) for the long and the short isoforms (see Experimental Procedures). The lists of ASEs (42,000–45,000 ASEs) were filtered to keep only data with at least two replicates for both types of HCC and normal hepatic tissue. The ASEs that differed between HCC tissues and normal hepatic tissue with a P-value of less than 0.05 were conserved. To ensure higher stringency, the ASEs were further filtered with a cutoff Q-value (false-discovery rate) of less than 0.05. From these events, only those with a difference higher than 10 % in |Delta PSI| were considered biologically relevant. Using such an approach, we identified 3250 primary transcripts belonging to 2051 genes for which the AS pattern was significantly modified in patients with HBV-associated HCC (Q ≤0.05, |Delta PSI| ≥10) (Fig. 1c). Moreover, the AS patterns of 1380 primary transcripts belonging to 907 genes were significantly modified in patients with HCV-associated HCC. The complete list of the differential ASEs associated with HBV- and HCV-associated HCC is provided in Additional file 1. The list includes six of the previously identified aberrantly spliced isoforms found in HCC in previous studies (*DNMT3b, AURKB, UBE3B, MDM2, KLF6,* and *TP73*). RNA-Seq data were not available for the two other previously identified examples of aberrantly-spliced genes detected in HCC (*CDH17* and *HUGL1*).

**Fig. 2:**
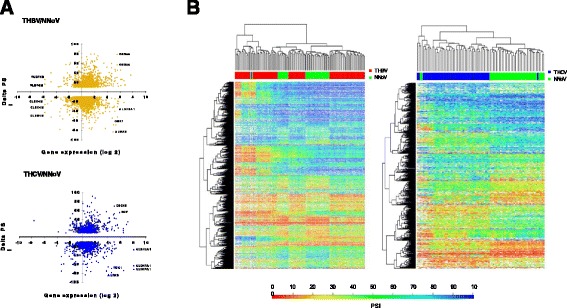
Global profiling of alternative splicing event modifications in HBV- and HCV-associated HCC. **a** Alternative splicing event modifications in HBV-associated HCC (THBV/NNoV) and HCV-associated HCC (THCV/NNoV) are presented with their associated changes in gene expression. The graphs represent the relation between the difference of splicing for each hepatitis-associated HCC compared with healthy non-viral tissues, and the difference in gene expression for each of these alternative splicing event modifications. **b** Heatmap representations of isoform ratios (PSI values) for all tissues analyzed in the current study. The first heat map shows PSI values for HBV-associated HCC. HBV-associated HCC tissues (THBV) are shown in red, and the comparative healthy tissues are shown in green (NNoV). The second heat map shows PSI values for HCV-associated HCC. HCV-associated HCC tissues (THCV) are shown in blue, and the comparative healthy tissues are shown in green (NNoV)

**Fig. 3:**
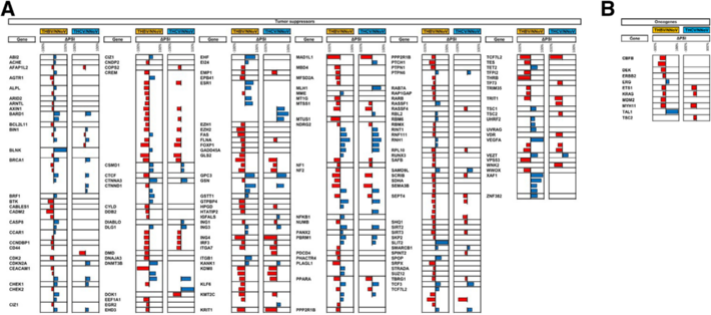
AS modifications in transcripts encoded by tumor suppressors and oncogenes in HBV- and HCV-associated HCC. **a** Alterations in the AS patterns of transcripts encoded by tumor suppressors in HBV- and HCV-associated HCC. Some transcripts have multiple ASEs that are modified. Red bars indicate negative Delta PSI values, and blue bars represent positive Delta PSI values. **b** Alterations in the AS patterns of transcripts encoded by oncogenes in HBV- and HCV-associated HCC. The tumor suppressors and oncogenes were selected based on the TSGene [64] and allOnco databases (http://www.bushmanlab.org/links/genelists)

Our analysis revealed that the expression of a vast number of cellular transcripts was modified in patients with HBV- and HCV-associated HCCs as compared to normal healthy tissues (Fig. 2a–b). In the case of HBV-associated HCC, 361 transcripts had |Delta PSI| values higher than 30 % (Additional file 1: Figure S1A), while 200 transcripts had |Delta PSI| values greater than 30 % for HCV-associated HCC. To identify cellular pathways for the differential ASEs associated with HBV- and HCV-associated HCC, we performed a functional enrichment analysis of the genes for which AS is modified. In both cases, the analysis revealed significantly enriched (Q ≤0.05) terms in both metabolic process (28.6 and 27.4 % for HBV- and HCV-associated HCC, respectively) and developmental process (6.6 and 7.1 % for HBV- and HCV-associated HCC, respectively) (Additional file 1: Figure S2A). Indeed, a significant number of the modified ASEs are encoded by genes with important roles in metabolism. For instance, alterations in the splicing patterns of BCAT2, which encodes a branched chain aminotransferase found in mitochondria, DAP3, which helps in protein synthesis within the mitochondrion, and ECHDC2, which is involved in fatty acid biosynthesis, were found to be present both in HBV- and HCV-associated HCC. A large number of these proteins appear to interact either directly or indirectly with each other (Additional file 1: Figure S2B). Through manual curation of functional annotations, we also identified numerous modifications in the AS patterns of genes potentially involved in oncogenic transformation. We identified many transcription factors, receptors, tumor suppressors, and kinases for which AS is significantly modified in HBV- and HCV-associated HCC (Additional file 1: Table S1). For example, HBV-associated HCC displayed alterations in the AS patterns of 138 tumor suppressors, 156 transcription factors, 73 kinases, and 132 receptors. Some of the dysregulated ASEs that were identified include KRAS, a member of the small GTPase superfamily implicated in various pathologies (such as lung adenocarcinoma, pancreatic cancer and colorectal carcinoma), MDM2, a nuclear-localized E3 ubiquitin ligase which can promote tumor formation by targeting tumor suppressor proteins (such as p53) for proteasomal degradation, and BRCA1 which encodes a nuclear phosphoprotein that plays a role in maintaining genomic stability, also acts as a tumor suppressor and is frequently mutated in breast and ovarian cancers. Interestingly, some of these modifications are found both in HBV- and HCV-associated HCC while others are unique to the respective virus-associated HCC (Fig. 3a–b and Additional file 1: Figure S3A–B). It should be noted that we found no evidence for correlation between the presence of one or more splice variants and clinical parameters (such as patient survival).

### Characterization of the ASEs that are modified in HCC

We next compared the profiles of the ASEs that are altered in HBV- and HCV-associated HCC. The vast majority of the ASEs that are modified in HCC were found at a level of one splicing event per gene (Additional file 1: Figure S1B). Analysis of the correlation between AS and gene expression indicated stable expression of most of the transcripts that are differentially spliced (Fig. 2a). Overall, we observed no clear association between the expression level and the cancer-specific splicing pattern, demonstrating that there is no relationship between the regulation of transcription and the control of alternative splicing in these cancer-specific genes.

Among the transcripts for which AS was significantly affected in HBV- and HCV-associated HCC, many ASEs we documented affect known protein domains. Additional file 1: Table S2 displays the consequences of the differentially spliced transcripts on protein function for 60 transcripts that are differentially spliced in HCC. Among the differential ASEs, at least two ASEs resulted in the loss of predicted nuclear localization signals (NLS). These are RINT1, which plays a role in cell cycle checkpoint control and is essential for telomere length control, and EHD3, a paralog of EHD1, a protein which is thought to play a role in the endocytosis of IGF1 receptors. Twenty other ASEs were predicted to disrupt known functionally critical protein domains. Most of these genes encode for proteins involved in metabolic processes. For instance, some of the dysregulated ASEs lead to protein domain loss in HADHA. This gene encodes the alpha subunit of the mitochondrial trifunctional protein, an enzyme which catalyzes the final three steps of mitochondrial beta-oxidation of long chain fatty acids, and ALDH2, the second enzyme of the major oxidative pathway of alcohol metabolism.

### Experimental validation on HCC tissues

**Fig. 4:**
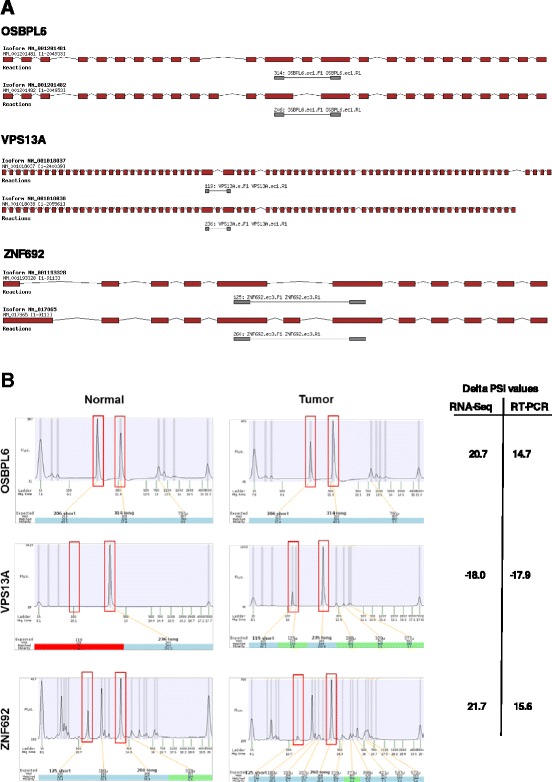
Validation of ASEs dysregulated in HCC. **a** Overview of the two isoforms encoded by *OSBPL6, VPS13A* and *ZNF692* genes. Exons are depicted in red and the intervening introns are shown as thin black lines (not to scale). The primers used to detect the isoforms by RT-PCR assays are shown in gray and the sizes of the expected amplicons are also indicated. **b** cDNA obtained from tissues were analyzed by PCR using specific primers to detect both isoforms of the transcripts encoded by the *OSBPL6, VPS13A and ZNF692* genes. Capillary electrophoregrams of the PCR reactions are shown. The positions and the amplitude of the detected amplicons are highlighted by red boxes. The positions of the internal markers are also indicated. The Delta PSI values for both the RNA-Seq and experimental validation assays are shown on the right

We next sought to experimentally validate the results that were obtained from the RNA-Seq data. We used PCR analysis to experimentally validate the differential ASEs that were observed through RNA-seq studies. Using liver cDNA arrays from both healthy and cancerous tissues, we designed specific primers to allow detection of the various ASEs by PCR. Examples of differentially spliced transcripts are presented in Fig. 4a illustrating the modifications in isoform usage in transcripts encoded by three different genes (*OSBPL6, VPS13A, ZNF692*). In all three cases, the experimental Delta PSI values were similar to the values obtained from RNA-Seq analysis (Fig. 4b). The experimental Delta PSI values ranged from −17.9 (for the VPS13A transcript) to 15.6 (for the ZNF692 transcript). Overall, our results demonstrated that changes in AS levels revealed through transcriptome sequencing could also be detected by PCR analysis (Fig. 4b).

### Comparison between different types of HCC

**Fig. 5:**
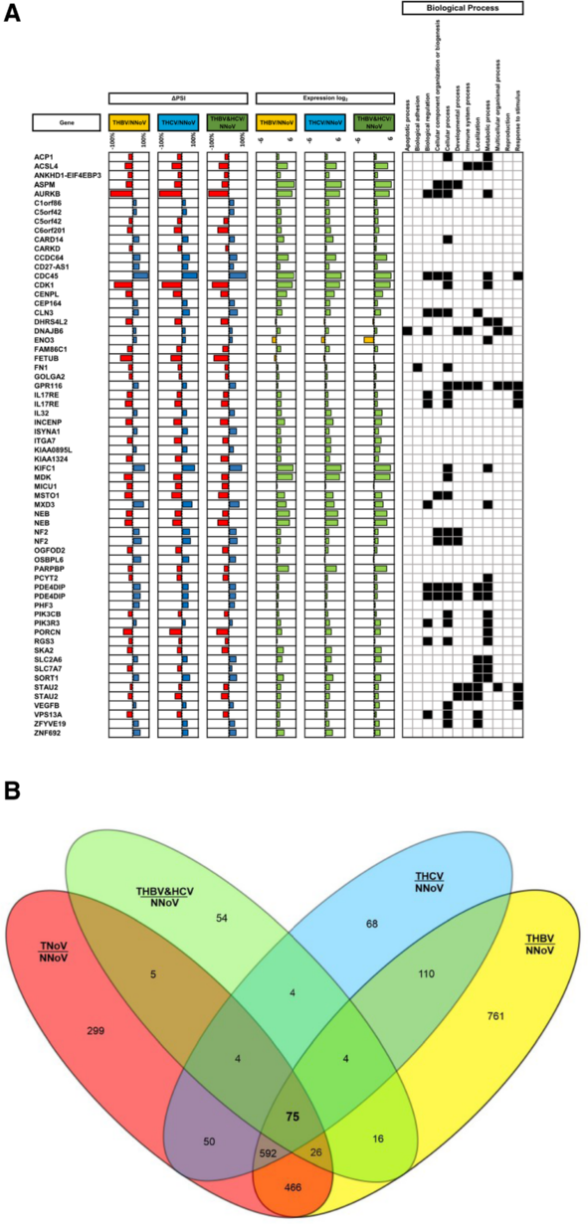
Comparison between HBV-, HCV-, HBV&HCV-, and non-viral-associated HCC. **a** The list displays the common differentially spliced transcripts for the various types of HCC with the corresponding Delta PSI values, the associated gene expression (in Log_2_), and the associated biological processes. **b** Comparison of the genes with dysregulated ASEs between HBV-, HCV-, HBV&HCV-, and virus-free HCC

The large size of the samples analyzed by high-throughput RNA sequencing allowed us to obtain data for the alterations to the global RNA splicing landscape of two other types of HCC, namely HCC associated with the co-presence of both HBV and HCV (THBV&HCV) and virus-free HCC (TNoV, Tumor no virus). Because of their shared modes of transmission, co-infection of HBV and HCV is not uncommon, particularly in countries with a high prevalence of HBV or HCV [19]. HBV and HCV co-infection results in more severe liver pathologies and in an enlarged risk of HCC than monoinfection [19]. While the number of HCC tissues associated with the presence of both HBV/HCV was relatively small in our samples collection (*n* = 3), our statistical analysis allowed us to identify 220 transcripts for which AS is modified in comparison to normal tissues (Additional file 1: Figures S1C, S4A, Tables S3–S6). Remarkably, 79 genes for which AS was modified were found to be common between the three types of virus-associated HCC (Fig. 5a). This raised the possibility that these common ASEs might be related to a general characteristic of cancerous tissues. We therefore analyzed the cellular AS patterns that are altered in virus-free HCC of 131 patients (TNoV; tumor, no virus), and we evaluated the changes in relative splice abundances. Using such an approach, we identified 1517 genes for which the AS pattern was significantly modified in virus-free HCC (Q ≤0.05, |Delta PSI| ≥10) (Additional file 1: Figures S1C, S4B, Tables S3-S6). Further analysis revealed that 75 of the 79 previously identified genes for which AS was modified in the three types of virus-associated HCC were also identified in virus-free HCC, thereby indicating that the modifications in AS found in those genes are associated with carcinogenic tissues. It should be noted that the AS of the four remaining genes were also found to be modified in virus-free HCC although they were not retained following the statistical analysis i.e. the Q-values were slightly higher than 0.05 and/or the |Delta PSI| were slightly lower than 10. Overall, our analysis allowed us to identify 761 unique transcripts for which AS is misregulated in HBV-associated HCC, while 68 are specific to HCV-associated HCC, 54 to HBV&HCV-associated HCC, and 299 to virus-free HCC (Fig. 5b, Additional file 1: Tables S7–S11).

### Expression levels and AS of RNA splicing factors

**Fig. 6:**
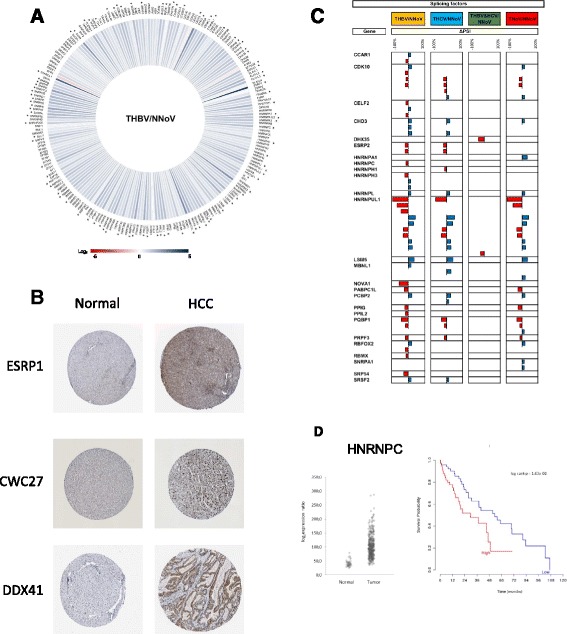
RNA splicing factors in HCC. **a** Iris Graph representing the expression profile of splicing factors for HBV-associated HCC. Differences in gene expression levels are shown on a logarithmic color scale (Log2), from red (negative changes in expression) to blue (increase in gene expression). **b** Expression of splicing factors in both normal liver tissues and hepatocellular carcinomas. These images were extracted from the Human Protein Atlas database, according to its academic usage permission (see Ref. [65] and www.proteinatlas.org). The images show the results of immunohistochemical staining using specific antibodies (complete list and experimental protocol found at www.proteinatlas.org), followed by detection with horseradish peroxidase. Immunohistochemical staining was performed on both normal and HCC tissues for three different splicing factors (ESRP1, CWC27, and DDX41). **c** Misregulation of splicing factors alternative splicing in HBV- (THBV/NNoV, yellow), HCV- (THCV/NNoV, *blue*), HBV&HCV- (THBV&HCV/NNoV, *green*) and virus-free HCC (TNoV/NNoV, *red*). The delta PSI values are represented in red (negative delta PSI values) and in blue (positive delta PSI values). **d** Expression levels of hnRNPC in patients with or without tumors (*left panel*). Kaplan–Meier overall survival curve (right panel) for HCC patients expressing high (*red*) or low (*blue*) levels of hnRNPC. HNRNPC transcript level was negatively correlated with overall survival

The precise mechanism which leads to a modification of the AS landscape in HCC is unknown at the moment. Changes in splice site choice generally arise from variations in the assembly of the spliceosome or by altering the binding of splicing factors to the RNA transcripts [20]. Although splicing is controlled by a large set of splicing factors, dysregulated expression of individual splicing factors has been shown to frequently result in aberrant splicing [21]. Taking these findings into consideration, we thus monitored both the expression levels and the changes in splicing patterns of RNA encoding for splicing factors and spliceosomal proteins. As shown in Fig. 6a, the expression of some splicing factors and spliceosomal proteins is indeed affected in HCC. The expression of 94 proteins involved in splicing was modulated by more than 2-fold in HBV-associated HCC. Interestingly, the expression of ESRP1, a splicing factor known to regulate diverse types of alternative splicing events [22], was increased by more than 21-fold in HBV-associated HCC. Very similar expression profiles were also found in HCV-, HBV&HCV-associated HCC and virus-free HCC (Additional file 1: Figure S5). Immunonohistochemical staining confirmed the over-expression of various splicing factors in HCC tissues, as compared to normal liver tissues, including ESRP1, CWC27, and DDX41 (Fig. 6b).

Modifications to the AS patterns of transcripts encoding proteins involved in splicing (splicing factors and proteins of the spliceosome) was also investigated. Our study identified many splicing factors that were differentially spliced upon in HCC (Fig. 6c). In the case of HBV-associated HCC, the AS patterns of 26 splicing factors were significantly modified. One example is RBFOX2, which encodes an RNA-binding protein that is thought to be a key regulator of alternative exon splicing. Other examples include CELF2, which regulates pre-mRNA alternative splicing and may also be involved in mRNA editing, and translation, and MBNL1, which can also regulate splicing on specific pre-mRNA targets. Modifications to both the expression level and AS of splicing factors likely contribute to the observed changes in the cellular AS landscape of HCC.

**Fig. 7:**
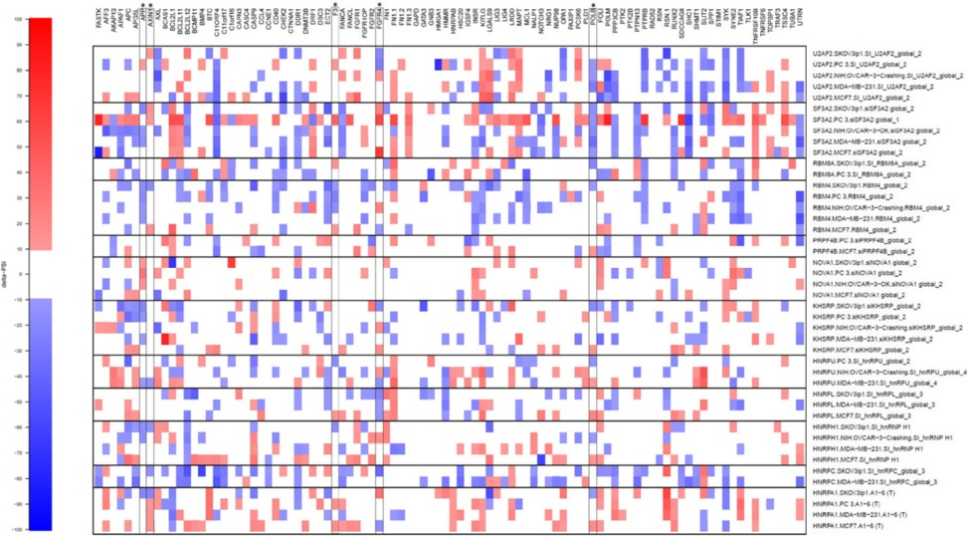
Modifications to AS of 96 transcripts in response to knockdown of splicing factors with specific siRNAs. Using specific siRNAs, twelve splicing factors (U2AF2, SF3A2, RBM8A, RBM4, PRPF4B, NOVA1, KHSRP, HNRPU, HNRPL, HNRPH1, HNRPC, and HNRPA1) were individually knocked-down in different cell lines to assess their implication in splicing of 96 differents transcripts. Individual knockdowns and ASEs are shown to indicate which knockdowns caused a shift in alternative splicing in various cell lines (PC-3, SKOV3, NIH:OVCAR-3, MDA-MB-231, MCF7). Each column represents a distinct knockdown performed with specific siRNAs. The changes in PSI values are displayed. The map depicts the changes in PSI values in a color-coded scale. White areas indicate no shifts. Asterisks indicate transcripts for which AS was altered in HBV-associated HCC

We further investigated the implication of splicing factor levels on AS by using RNA interference and a reverse transcription-PCR screening platform to examine the roles of splicing factors in different cell lines [23]. We selected various splicing factors which were over- or under-expressed in HBV- and HCV-associated HCC. These splicing factors were each targeted with specific siRNAs, and we carried out a loss-of-function study in various cell lines (PC-3, SKOV3, NIH:OVCAR-3, MDA-MB-231, MCF7). In the case of HBV-associated HCC, specific siRNAs were designed against splicing factors U2AF2, SF3A2, RBM8A, RBM4, PRPF4B, NOVA1, KHSRP, HNRPU, HNRPL, HNRPH1, HNRPC, and HNRPA1. Depletion efficiencies were evaluated 96 h posttransfection by Western blotting and/or quantitative RT-PCR assays. All assays indicated that depletion had been achieved (data not shown). We selected 96 transcripts that belong to a subset of apoptotic genes because the functional consequences of alternative splicing on apoptosis have previously been clearly established [24]. Five of these transcripts (APP, AXIN1, F3, FGFR4, and POLB) were of particular interest since their AS was altered in HBV-associated HCC. The results are depicted in Fig. 7 and show that the impact of the individual knockdowns varies considerably. For instance, the knockdown of SF3A2 has a definitive impact on the AS pattern of the BCL2L1, C11ORF4, DRF1, FANCA, FN1.3, POLB, PTPN13, SHC1, and TNFRSF10B transcripts in all cell lines tested. However, the same knockdown has no impact on the AS profile of numerous transcripts such as BCMP11, CCL4, CTNNA1, FGFR1, FGFR2, FN1.2, GAPD, GATA3, GNB3, HNRPAB, HSC20, PAXIP1, PTK2, PTK2B, RAD52, RSN, SHMT1, STM1, and TLK1 transcripts. We conclude that the expression of individual splicing factors has an impact on AS profiles, but cannot explain all the changes observed. The impact of the individual knockdowns on AS was also analyzed for splicing factors which were over- or under-expressed in HCV-associated HCC (Additional file 1: Figure S6). As observed previously [23], our results demonstrate that targets for individual RNA splicing factors can vary in different cellular contexts.

### Expression pattern of hnRNPC significantly correlates with patient survival

We next investigated the relationship between the mRNA expression levels of proteins involved in RNA splicing and patient survival. A substantial number of HCC biomarkers with potential prognostic significance have been identified in recent decades and have shown promising results [25]. Kaplan-Meier analysis was performed to evaluate overall survival for patients as a function of the mRNA expression levels of proteins involved in splicing. Of all the proteins involved in splicing for which the expression level was modulated by more than 2-fold in HCC, the expression level of hnRNPC, a regulator of mRNA splicing, was the only one showing correlation with patient survival (Fig. 6d). A high level of expression of hnRNPC was associated with reduced patient survival (*p* < 0.02 by Cox regression assay). No significant correlations were found between high-levels of hnRNPC expression and clinicopathological features, including age, gender, and tumor stage. Interestingly, the previous depletion assays (Fig. 7) indicated that a decrease in hnRNP C expression affected 20 % (19/96) of the ASEs tested in at least two cell lines, thereby demonstrating the importance of this protein in the regulation of AS.

### Expression of HBx alters cellular AS

**Fig. 8:**
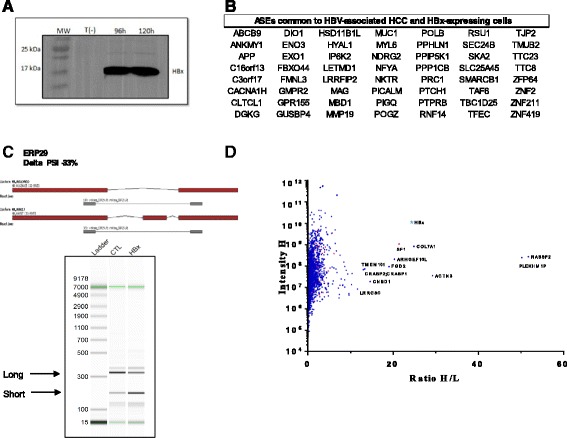
Involvement of the HBx protein from HBV in AS. **a** Immunoblotting analysis using anti-HA antibody for the detection of HBx-HA protein from the cell lysates isolated after 5 days of transfection with pLenti6V5A-HBx. Control untransfected cells (T(−)) were also used in this assay. **b** List of ASEs common to HBV-associated HCC and HBx-expressing cells. **c** Example of an ASE modified following the expression of HBx. Overview of the two isoforms encoded by *ENO3* gene. Exons are depicted in red and the intervening introns are shown as thin black lines (not to scale). The primers used to detect the isoforms by RT-PCR assays are shown in gray and the sizes of the expected amplicons are also indicated (top panel). RT-PCR reactions were performed on control cells (T(−)) and cells expressing HBx (HBx) using specific primers to detect both isoforms of the transcripts encoded by the *ENO3* gene. Capillary electrophoresis was performed and an image of the detected reaction products is shown (lower panel). The positions of the expected amplicons are indicated by arrows. **d** Mass spectrometry analysis of cellular proteins interacting with HBx. The average H/L ratios of the HBx affinity purification-mass spectrometry experiments were plotted versus the total intensities. The red dots indicate cellular proteins involved in RNA splicing

Although changes in splicing patterns are features of cancer development/progression, it whether these changes are the source or the consequence of a malignant phenotype remains unknown. We therefore investigated the attractive possibility that the presence of a viral protein could contribute to modifications in AS of virus-associated HCC. We focused on the HBx protein of HBV since currently available evidence supports a role for this protein in the pathogenesis of HBV-associated HCC. HBx has been shown to promote cell cycle progression, deactivate negative growth regulators, and prevent the expression of tumor suppressor genes and senescence-related factors [26]. The HBx protein (harboring a HA epitope) was expressed in HEK293T cells and detected by SDS-PAGE followed with anti-HA immunoblotting (Fig. 8a). HEK293T cells were chosen because of their high transfection efficiency and their extensive use in RNA splicing studies [27–30]. We next relied on a high-throughput reverse transcription-PCR (RT-PCR)-based platform for splicing annotation, to examine AS in 1200 cancer-associated genes [31]. Once again, the relative abundance of ASEs was expressed as PSI values. Our results demonstrated that the AS patterns of 56 cancer-related genes which were modified in HBV-associated HCC were also significantly modified upon HBx expression (Fig. 8b). An example of modified AS pattern modified upon HBx expression is provided in Fig. 8c.

The observation that the expression of HBx leads to modifications in the AS profile of cellular genes led us to investigate the ability of HBx to interact with cellular proteins involved in splicing. We used a quantitative proteomics-based approach relying on stable isotope labeling of amino acids in cell culture (SILAC) coupled to mass spectrometry (LC-MS/MS) to identify cellular proteins that interact with HBx. We used SILAC based on three different sets of conditions in order to detect cellular proteins that bind to HBx in the absence of RNA/DNA (see Methods). Cell lysates were incubated with anti-HA antibodies and bound proteins were precipitated, washed, eluted, trypsinized, and subjected to MS/MS. Potential contaminants and non-specific binders were considered using the CRAPOME database (Contaminant Repository for Affinity Purification and Mass Spectrometry) [32]. Using such a strategy, our quantitative mass spectrometry analysis demonstrated that SF1, a splicing factor which is necessary in the early phases of spliceosome assembly, was highly associated with HBx (Fig. 8d). No other cellular protein involved in RNA splicing was strongly associated with HBx. The role of this interaction in the regulation of AS in HBV-associated HCC will need to be further investigated in future studies.

## Discussion

In the current study, we present the first large-scale screen of HCC–associated ASEs. The study demonstrated that the AS patterns of numerous cellular transcripts are modified in HCC. Moreover, these data allowed us to identify unique signatures of genes for which AS is misregulated in the different types of HCC. Analysis of the AS landscape of the various types of HCC revealed numerous potential HCC–specific markers, which significantly increases the number of biomarkers that can presently be identified by current expression profiling approaches. We believe that our study could lead to the identification of molecular candidates for diagnostics and/or targetable pathways in HCC. Currently, α-fetoprotein (AFP) together with pathology and iconography detection are routinely used in early clinical diagnosis for liver cancer [33]. However, the widely used marker AFP does not always yield satisfactory results in the early diagnosis of HCC, particularly in the case of AFP-negative HCC, thereby limiting the universality of its application [33]. The use of other HCC markers (such as GPC3 and TGF-β1) is presently being explored [34], which is expected to improve early diagnostic rate. Undoubtedly, future developments in molecular genetics and proteomic analysis should lead to the identification of other useful HCC-specific markers. The current identification of unique signatures for genes in which AS is misregulated in the different types of HCC constitutes a step toward that goal.

In order to meet the augmented requirements of proliferation, cancer cells frequently display important changes in pathways of energy metabolism and nutrient uptake [35]. Oncogenic transformation often results in an increase in both bioenergetic potential and the nutrient uptake in a cell-autonomous fashion [36]. The fact that metabolic enzymes such as succinate dehydrogenase (SDH) and fumarase (FH) function as tumor suppressors in human cells suggests that metabolic dysregulation can be an initiating event in cancer [36]. The recent discovery of activating mutations in isocitrate dehydrogenase (IDH1) in glioblastoma demonstrates that an activating mutation in a metabolic enzyme are selected during carcinogenesis [37]. The emergence of metabolic enzymes acting as important regulators of cancer cell growth highly suggests that metabolic control is a critical factor in carcinogenesis. In the present study, many transcripts that were differentially spliced in HCC samples are known to play critical roles in cell metabolism. For example, the expression of the full-length ALDH2 transcript was found to be significantly reduced and replaced by a shorter form that is predicted to encode a truncated Aldh2 protein with a loss of a portion of the aldehyde dehydrogenase domain. Aldehyde dehydrogenase is a key enzyme involved in the major oxidative pathway of alcohol metabolism. Interestingly, the enlarged exposure to acetaldehyde in patients with a catalytically inactive form of Aldh2 has previously been shown to confer an increase susceptibility to many types of cancer, including esophageal cancer [38]. Another example of a gene involved in metabolism for which AS is modified in HCC is *HADHA,* which encodes the alpha subunit of the mitochondrial trifunctional protein catalyzing the 3-hydroxyacyl-CoA dehydrogenase and enoyl-CoA hydratase activities. These steps are involved in mitochondrial beta-oxidation of long chain fatty acids. Our results showed that the expression of the full-length HADHA transcript was found to be significantly reduced and replaced by a shorter form that is predicted to encode a truncated Hadha protein with a loss of a portion of the enoyl-CoA domain. Interestingly, a link between *HADHA* and cancer has previously been observed in breast cancer where the *HADHA* gene was significantly under-expressed in cancerous tissues, especially in tumors with estrogen receptor-negative status [39].

In comparison with normal tissues, a substantial number of RNA splicing factors have been found to be upregulated or downregulated in various cancers. As changes in the concentration of these factors have been shown to modify the selection of splice sites [40], it is predicted that the abnormally expressed splicing factors found in cancerous cells induce the production of mRNA isoforms that are either nonexistent or less abundant in normal cells. A major challenge will be to determine if these modifications contribute directly to carcinogenesis, or whether they constitute one of the many processes that are modified in cancer cells. The demonstration that overexpression of the Serine/Arginine-Rich Splicing Factor 1 (SRSF1, also known as SF2/ASF) can trigger malignant transformation suggests that the abnormal expression of proteins involved in splicing can contribute to carcinogenesis [41]. In addition, the expression SRSF1 has been shown to be increased in several tumor types [42] and fibroblasts overexpressing SRSF1 caused tumor formation when injected into mice [41]. In the current study, many changes in the expression levels and/or splicing patterns of splicing factors were detected in HCC samples. Interestingly, our data demonstrated that the transcript encoded by *ESRP1* was found to be significantly over-expressed in all types of HCC investigated (32-fold increase in HBV-associated HCC). How the altered expression dynamics of *ESRP1* (and other splicing regulators) contribute to AS homeostasis remains to be examined. Interestingly, a previous report identified ASEs regulated by Esrp1 using RNA silencing technology in a human epithelial cell line [43]. Using such a strategy, the authors identified 148 alternative splicing events in a total of 134 different genes that were regulated by Esrp1 [43]. In the present study, the splicing of 32 of these Esrp1-regulated transcripts are also found to be differentially spliced in HBV-associated HCC suggesting, at least in part, that some of the observed splicing alterations observed in HCC could be related to Esrp1 over-expression. In the case of HCV-associated HCC and virus-free HCC, the splicing of 17 and 20 of these Esrp1-regulated transcripts were also found to be differentially spliced, respectively.

AS has been shown to add an additional layer of regulation that functions in concert with transcriptional alterations during the epithelial-to-mesenchymal transition (EMT) [43]. EMT is an important and reversible process during the development/differentiation of multiple organs and has been implicated in cancer progression and metastasis [44]. It involves reorganization of the actin cytoskeleton, loss of cell–cell adhesion, the acquisition of increased cell motility, and changes in the transcriptional profile of epithelial cells (such as downregulation of E-cadherin and upregulation of vimentin and N-cadherin [45]. Moreover, previous studies have shown that a downregulation of epithelial-specific splicing regulators (ESRP1 and ESRP2) occurs in cells which undergo EMT [46]. Down-regulation of ESRP1 and ESRP2 has also been closely associated with a motile phenotype of cancer cells [47]. EMT is a crucial event in HCC progression which causes an increase in malignancy of hepatocytes associated with tumor cell invasion and metastasis [48]. For HCC, intrahepatic metastasis is most frequent as the hepatic microenvironment displays a dense vasculature and is the competent milieu for HCC cells in the metastasis cascade [48]. In the current study, expression of ESRP1 was significantly increased in HCC as evidenced from both RNA-Seq and immunohistochemical analyses. This finding was unexpected since downregulation of epithelial-specific splicing regulators is usually associated with cells undergoing EMT [46]. The exact significance of this result is not understood at the moment. In HCC, EMT likely occurs at the leading edge of tumor tissues under the particular control of extrinsic factors derived from the tumor microenvironment in a paracrine fashion [46]. As such, EMT is traditionally associated with highly metastatic HCC. Moreover, it should be noted that both the epithelial and mesenchymal cell phenotypes are not static. Indeed, both cell types have the capacity to undergo important cellular transformations (such as EMT and MET: mesenchymal-to-epithelial transition) that can alter both cellular morphology and behavior [43]. The MET-EMT cycle, along with differences between primary HCC and metastatic HCC, might also explain why no significant evidence for gene over-expression of traditional mesenchymal markers (such as vimentin or N-cadherin) or repression of epithelial ones (e.g., E-cadherin) was found in tissues from HCC patients in the TCGA database.

Tumor markers based on the expression of specific proteins that can be used in the prognosis of HCC have been identified during the past few years, although consensus has still not been reached [25]. More recently, an important number of profiles based on the expression levels of mRNAs have been used as predictors of prognosis in various cancers [49]. For instance, the mRNAs levels of hnRNP K, a member of the hnRNP family of proteins which are involved in transcription, RNA splicing, and translation, has previously been shown to be aberrantly increased in numerous cancers [50, 51]. It was reported that high-level hnRNP K expression is correlated with poorer overall survival among patients with nasopharyngeal carcinoma (NPC) and prostate cancer [50, 51]. High levels of expression of another member of the family, namely hnRNP D, has also been linked to reduced survival in oral cancer [52]. In the current study, a high level of mRNA expression of hnRNPC was also associated with reduced patient survival in HCC. Clearly, additional studies will need to be performed to see if such an observation can eventually have any clinical application. Nonetheless, validation of potential tumor markers such as hnRNPC will continue to be a major focus of HCC research in the next few years. Indeed, improvements in early diagnosis are still needed since only 30 % of patients with HCC are candidates for potentially curative treatments at the time of diagnosis [53]. We believe that some RNA splicing isoforms markers could eventually be used as biomarkers. Ultimately, these could serve as diagnostic or prognostic tool by detecting their presence in cancer cells or in the fluids of patients.

Large-scale studies are starting to reveal the extent of modifications that occur in AS in different types of cancer [14]. Although the precise mechanism which leads to a modification of the AS landscape in HCC is unknown at the moment, we demonstrated that the expression of the HBV HBx protein can result in modifications in the AS profiles of cellular genes. Our results showed that the AS patterns of 56 cancer-related genes which were modified in HBV-associated HCC were also significantly altered upon HBx expression, thereby suggesting a potential mechanism by which a viral infection can alter cellular AS profiles. Many direct and/or indirect molecular mechanisms by which the expression of a viral protein such as HBx can lead to a modification of the cellular AS landscape can be envisaged. However, the demonstration that HBx can interact with splicing factor SF1 is clearly a step towards identifying the steps required for the extensive modifications of AS observed in HCC. Interestingly, strategies to modulate AS by splice-switching oligonucleotides in order to correct aberrant ASEs, and/or to induce expression of therapeutic splice variants are now being developed [17, 54]. It is tempting to speculate that such a strategy could be applied to HCC. The current identification of extensive changes in the cellular AS landscape in HCC likely represents a first step toward the development of anticancerous agents based on the AS modifications identified in HCC. The development of highly specific molecular tools, such as splice-switching oligonucleotides, which could precisely alter the proportion of splice variants will also be critical to assess the function of these splice variants.

## Conclusions

The present analysis provided a comprehensive portrait of global changes in the RNA splicing signatures that occur in HCC. These data also allowed the identification of unique signatures of genes for which AS is misregulated in different types of HCC which could lead to the identification of new molecular candidates for diagnostics and/or targetable pathways in HCC.

## Methods

### RNA-seq data analysis

RNA-Seq samples were provided by the CGHub data portal (https://cghub.ucsc.edu/). As only BAM files were available at the time the data was obtained, a custom script was used to extract read information from the given alignment files and generate valid FASTQ files for re-alignment (two FASTQ files with the reads in the same order such that the aligner can align them in pairs). Sequence reads were aligned on a transcriptome reference sequence database (UCSCGene Hg19 using Bowtie v2 aligner (default parameters) and associated gene isoforms quantified in transcript-per-million (TPM) using RSEM for each sequenced sample [55]. Alternative splicing events were automatically detected and quantified using the percent-spliced-in (PSI, Ψ) metric based on isoform expression TPM for the long (L) and the short (S) forms using equation below:

$$ \Psi = \frac{\mathrm{L}}{\mathrm{L} + \mathrm{S}} $$

Genes with one single isoform or no Human Genome Organisation (HUGO) ID were not considered for further analysis.

### Ethics, consent, and permissions

Not applicable. RNA-Seq data were obtained from the TCGA Portal (https://tcga-data.nci.nih.gov/tcga/).

### Gene expression analysis

The gene list was initially filtered to keep only data present in at least two replicates for both virus-associated HCC and normal hepatic tissue. The amount of transcript in each sample was calculated in transcripts per million (TPM) from the transcript-estimated read counts which were provided by TCGA and the isoform lengths from the UCSC (June 2011) annotation. To ensure higher reproducibility, only genes with expression levels higher than one TPM in either dataset were conserved. Fold changes in base 2 logarithm were then calculated between both virus-associated HCC and normal hepatic tissue average TPM. Q-values were calculated to take into account multiple statistical hypothesis testing and results under 0.05 were considered significant.

### Alternative splicing analysis

The alternative splicing event (ASE) list was filtered to keep only data present in at least two replicates for both virus-associated HCC and normal hepatic tissue. Events with a P-value less than 0.05 were conserved. To ensure higher stringency, the ASEs were further filtered with a cutoff Q-value of less than 0.05. From these events, only those with a difference greater than 10 % in PSI were considered biologically relevant.

### Statistical analysis

Welch’s *t*-test (Student’s *t*-test with unequal sample sizes and unequal variances) was calculated through the GSL library (http://www.gnu.org/software/gsl/) integrated to Perl system analysis for gene expression and alternative splicing data. Also, false discovery rates were calculated with the Q-value package in R (https://cran.r-project.org/src/contrib/Archive/qvalue/) based on Storey and Tibshirani [56]. For all other analyses, Graph Pad Prism version 6.05 was used to run statistical analysis.

### Gene ontology analysis

Gene ontology analysis with PANTHER was performed using the database for annotation, visualization, and integrated discovery (DAVID) [57].

### String networks

Using the STRING database (version 10) [58], genes were submitted for generation of protein-protein interaction network from the *Homo sapiens* interactome. High-resolution evidence views were created and saved.

### Functional ASE prediction

Using the FAST-BD or EASANA suite, the splicing patterns of genes of interest were visualized. DNA sequences of representative transcripts presenting short and long isoforms were downloaded and translated into proteins using ExPASy translation tool [59]. Predicted proteins were then compared using Multalin (truncation and frameshift event) [60], PFAM (loss or appearance of a functional domain) [61], and NLS Mapper (loss or gain of nuclear localization signal) [62].

### PCR validation

Liver Cancer cDNA Arrays form Origene TissueScan plates (Rockville, MD) were assessed for the expression of the various transcripts using the manufacturer’s protocol. The plates contained cDNAs from 8 normal and 39 liver cancer tissues. All forward and reverse primers were individually resuspended to 20–100 μM in Tris-EDTA buffer (IDT) and diluted as a primer pair to 1 μM in RNase DNase-free water (IDT). PCR reactions were performed in 10 μl in 96 well plates on a CFX-96 thermocycler (BioRad). The following cycling conditions were used: 3 min at 95 °C; 50 cycles: 15 s at 95 °C, 30 s at 60 °C, 30 s at 72 °C. For every PCR run, control reactions performed in the absence of template were performed for each primer pair and these were consistently negative. The amplified products were analyzed by automated chip-based microcapillary electrophoresis on Caliper LC-90 instruments (Caliper LifeSciences). Amplicon sizing and relative quantitation were performed by the manufacturer’s software.

### Splicing factors knockdown

siRNAs, Western blot analysis, RT-PCR assays and bioinformatic analysis were performed as described before [23].

### Immunoprecipitation and mass spectrometry

HEK293T cells were grown in light (DMEM-R0K0), medium (DMEM-R6K4) or heavy media (DMEM-R10K8) containing the heavy isotopes of arginine and lysine supplemented with 10 % fetal bovine serum. Cells were transfected with pLenti6V5A-HBx to express the HBV HBx protein for five days. Untransfected control cells were also grown for 5 days. Cells were harvested separately, washed three times with PBS, and treated (heavy) or not (light and medium) with 20 mg/ml RNase A (Invitrogen) and 200 units DNase RQ1 (Promega) for two hours. Immunoprecipitation was performed using HA beads (Roche 11815016001) following the manufacturer’s instructions. LC-MS/MS was performed as described previously [63].

## Additional file

Additional file 1: Table S1. Protein families encoded by transcripts that are differentially spliced in various types of HCC. **Table S2.** Bioinformatical prediction of functional changes caused by some of ASEs identified. **Table S3.** List of tumor suppressors for which AS is dysregulated in various types of HCC. **Table S4.** List of oncogenes for which AS is dysregulated in various types of HCC. **Table S5.** List of kinases for which AS is dysregulated in various types of HCC. **Table S6.** List of transcription factors for which AS is dysregulated in various types of HCC. **Table S7.** List of genes for which AS is dysregulated in all types of HCC. **Table S8.** List of genes uniquely dysregulated in HBV-associated HCC. **Table S9.** List of genes uniquely dysregulated in HCV-associated HCC. **Table S10.** List of genes uniquely dysregulated in HBV&HCV-associated HCC. **Table S11.** List of genes uniquely dysregulated in virus-free HCC. **Figure S1.** Characterization of splicing mysregulation in HCC. **Figure S2.** Characterization of ASEs that are modified in HBV- and HCV-associated HCC. **Figure S3.** AS modifications in transcripts encoded by kinases and transcriptions factores in HBV- and HCV-associated HCC. **Figure S4.** Global profiling of ASE modifications in both HBV&HCV-associated HCC and virus-free-associated HCC. **Figure S5.** RNA splicing factors in HCC. **Figure S6.** Modifications to AS of 96 transcripts in response to knockdown of splicing factors with specific siRNAs (PDF 6675 kb)

## Abbreviations

AS: Alternative splicing
ASE: Alternative splicing event
HBV: Hepatitis B virus
HCC: Hepatocellular carcinoma
HCV: Hepatitis C virus
NNoV: Normal tissues, no virus
PSI: Percent-spliced-in
TCGA: The Cancer Genome Atlas
THBV: Tumors with HBV
THBV&HCV: Tumors with both HBV and HCV
THCV: Tumors with HCV
TNoV: Virus-free gastric hepatic tissues
TPM: Transcript-per-million
