# Supplementary material for: Global profiling of alternative RNA splicing events provides insights into molecular differences between various types of hepatocellular carcinoma
Source: BMC Genomics. 2016 Aug 26;17(1):683. doi: 10.1186/s12864-016-3029-z (PMC5002109; doi:10.1186/s12864-016-3029-z)
Supplement: Additional file 1: Table S1. — Protein families encoded by transcripts that are differentially spliced in various types of HCC. Table S2. Bioinformatical prediction of functional changes caused by some of ASEs identified. Table S3. List of tumor suppressors for which AS is dysregulated in various types of HCC. Table S4. List of oncogenes for which AS is dysregulated in various types of HCC. Table S5. List of kinases for which AS is dysregulated in various types of HCC. Table S6. List of transcription factors for which AS is dysregulated in various types of HCC. Table S7. List of genes for which AS is dysregulated in all types of HCC. Table S8. List of genes uniquely dysregulated in HBV-associated HCC. Table S9. List of genes uniquely dysregulated in HCV-associated HCC. Table S10. List of genes uniquely dysregulated in HBV&HCV-associated HCC. Table S11. List of genes uniquely dysregulated in virus-free HCC. Figure S1. Characterization of splicing mysregulation in HCC. Figure S2. Characterization of ASEs that are modified in HBV- and HCV-associated HCC. Figure S3. AS modifications in transcripts encoded by kinases and transcriptions factores in HBV- and HCV-associated HCC. Figure S4. Global profiling of ASE modifications in both HBV&HCV-associated HCC and virus-free-associated HCC. Figure S5. RNA splicing factors in HCC. Figure S6. Modifications to AS of 96 transcripts in response to knockdown of splicing factors with specific siRNAs (PDF 6675 kb) [file 12864_2016_3029_MOESM1_ESM.pdf]

Global profiling of alternative RNA splicing events provides insights into molecular differences between various types of hepatocellular carcinoma.

Marie-Pier Tremblay, Victoria E. S. Armero, Andréa Allaire, Simon Boudreault, Mathieu Durand, Elvy Lapointe, Philippe Thibault, Maude Tremblay-Létourneau, Jean-Pierre Perreault, Michelle S. Scott, Martin Bisaillon

SUPPLEMENTAL DATA

Supplementary table 1 – Protein families encoded by transcripts that are differentially spliced in various types of HCC

	THBV/NNoV		THCV/NNoV		THBV&HCV/NNoV		TNoV/NNoV	
**Protein classification**	**Freq.**	**Percent.**	**Freq.**	**Percent.**	**Freq.**	**Percent.**	**Freq.**	**Percent.**
Extracellular matrix protein (PC00102)	29	1.5%	12	1.4%	1	0.6%	18	1.2%
Protease (PC00190)	50	2.5%	18	2.0%	5	3.0%	38	2.5%
Cytoskeletal protein (PC00085)	100	5.1%	53	6.0%	10	6.0%	88	5.7%
Transporter (PC00227)	105	5.3%	56	6.3%	7	4.2%	91	5.9%
Transmembrane receptor regulatory/adaptor protein (PC00226)	4	0.2%	3	0.3%	1	0.6%	4	0.3%
Transferase (PC00220)	159	8.0%	64	7.2%	11	6.6%	121	7.8%
Oxidoreductase (PC00176)	68	3.4%	27	3.1%	5	3.0%	60	3.9%
Lyase (PC00144)	27	1.4%	11	1.2%	2	1.2%	16	1.0%
Cell adhesion molecule (PC00069)	52	2.6%	20	2.3%	3	1.8%	44	2.9%
Ligase (PC00142)	54	2.7%	32	3.6%	7	4.2%	49	3.2%
Nucleic acid binding (PC00171)	225	11.4%	114	12.9%	18	10.8%	173	11.2%
Signaling molecule (PC00207)	101	5.1%	39	4.4%	11	6.6%	61	4.0%
Enzyme modulator (PC00095)	147	7.4%	74	8.4%	16	9.6%	103	6.7%
Calcium-binding protein (PC00060)	40	2.0%	19	2.2%	5	3.0%	31	2.0%
Defense/immunity protein (PC00090)	63	3.2%	24	2.7%	4	2.4%	49	3.2%
Hydrolase (PC00121)	187	9.5%	77	8.7%	15	9.0%	144	9.3%
Transfer/carrier protein (PC00219)	39	2.0%	22	2.5%	2	1.2%	34	2.2%
Membrane traffic protein (PC00150)	54	2.7%	22	2.5%	7	4.2%	36	2.3%
Phosphatase (PC00181)	45	2.3%	17	1.9%	3	1.8%	38	2.5%
Transcription factor (PC00218)	156	7.9%	67	7.6%	15	9.0%	128	8.3%
Chaperone (PC00072)	15	0.8%	2	0.2%	0	0.0%	4	0.3%
Cell junction protein (PC00070)	10	0.5%	9	1.0%	2	1.2%	10	0.6%
Surfactant (PC00212)	6	0.3%	4	0.5%	0	0.0%	4	0.3%
Structural protein (PC00211)	9	0.5%	5	0.6%	1	0.6%	9	0.6%
Receptor (PC00197)	132	6.7%	53	6.0%	8	4.8%	112	7.3%
Kinase (PC00137)	73	3.7%	32	3.6%	6	3.6%	61	4.0%
Storage protein (PC00210)	5	0.3%	1	0.1%	0	0.0%	4	0.3%
Isomerase (PC00135)	20	1.0%	5	0.6%	1	0.6%	11	0.7%
Splicing factor	23	1.16%	13	1.6%	2	1.2%	15	1.0%
Tumor suppressor	138	6.7%	63	3.0%	10	6.0%	109	7.1%
Oncogene	9	0.5%	4	0.5%	1	0.6%	5	0.3%

Supplementary table 2 - Bioinformatical prediction of functional changes caused by some of ASEs identified. (Negative delta-PSI means that the short isoform is favored and positive delta-PSI means that the long isoform is favored)

Gene	Delta PSI				Event type	Predicted residue variation (AA)	Predicted functional consequence
	THBV/NNoV	THCV/NNoV	THBV&HCV/NNoV	TNoV/NNoV			
**A2M**	-13,1	N/S	N/S	N/S	exon-skipping	-150	Loss of signal peptide, loss of part of MG2 domain
**AAK1**	-10,3	N/S	N/S	N/S	alternate-3p	-1	Unknown/No predicted functional change
**ABCC10**	-15,1	N/S	N/S	-17,0	alternate-3p	28	Gain of signal peptide
**ACTA2**	-22,2	N/S	N/S	N/S	exon-skipping	-45	Loss of part of actin domain
**ACVRL1**	-15,5	N/S	N/S	N/S	multiple-exon-skipping	-174	Loss of a transmembrane domain, loss of a TGFb domain, loss of a tyrosine kinase domain
**ADCY5**	16,2	N/S	N/S	N/S	exon-skipping	8	Unknown/No predicted functional change
**ALDH1A3**	-16,4	N/S	N/S	N/S	multiple-exon-skipping	-107	Loss of a part of aldehyde dehydrogenase domain
**ALDH2**	-12,1	N/S	N/S	N/S	exon-skipping	-47	Loss of a part of aldehyde dehydrogenase domain
**AMDHD1**	-18,4	N/S	N/S	N/S	exon-skipping	-109	Loss a part of aminohydrolase family domain
**ANGPT1**	21,1	22,7	N/S	N/S	alternate-5p	1	Unknown/No predicted functional change
**ANKIB1**	-11,6	N/S	N/S	N/S	exon-skipping	-731	Loss of all domains (ankyrin repeat, ankyrin repeat 5, IBR domain (a half ring finger domain))
**ASPG**	-21,8	-19,7	N/S	-21,5	exon-skipping	-18	Unknown/No predicted functional change
**AXIN1**	-10,9		N/S	N/S	exon-skipping	-36	Unknown/No predicted functional change
**BUB1B**	N/S	-24,6	N/S	-20,5	alternate-3p	513	Gain a protein kinase domain and complete a Mad3/bub1 domain
**C16orf59**	29,0	31,9	N/S	33,6	alternate-3p	43	Complete the DUF (domain of unknown function) incomplete in the short isoform protein
**C5orf34**	30,5	25,6	N/S	27,6	exon-skipping	441	Complete the DUF4524 (domain of unknown function) incomplete in the short isoform protein
**CA13**	15,9	16,1	N/S	12,1	exon-skipping	262 AA protein to no protein	Loss of the protein with a carbonic anhydrase domain du to NMD

**CAND1**	-15,7	N/S	N/S		alternate-5p+alternate-3p	-490	Loss of transmembrane domains
**CENPL**	-25,4	-29,8	-34,3	-29,4	exon-skipping	-46	Unknown/No predicted functional change
**EHD3**	-12,7	N/S	N/S	N/S	multiple-exon-skipping	-360	Loss of 2 NLS, loss a part of dynamin domain
**EHF**	37,8	N/S	N/S	35,2	exon-skipping	23	Unknown/No predicted functional change
**GTPBP4**	-22,6	N/S	N/S	N/S	exon-skipping	-116	Unknown/No predicted functional change
**HADHA**	-18,1	N/S	N/S	N/S	exon-skipping	-87	Loss of a part of the enoyl-CoA hydratase/isomerase domain
**HAUS6**	-17,0	N/S	N/S	N/S	alternate-3p	-35	Unknown/No predicted functional change
**HBEGF**	-13,0	N/S	N/S	N/S	exon-skipping+alternate-5p+alternate-3p	208 AA protein to no protein	Loss of the protein with an EGF-like domain du to the exclusion of the only exon coding for protein
**HGF**	-18,5	-17,6	N/S	-14,9	alternate-3p	-5	Unknown/No predicted functional change
**IGFALS**	N/S	10,1	N/S	N/S	alternate-3p	38	Gain of transmembrane domains
**ITGB1**	24,7	N/S	N/S	N/S	exon-skipping	3	Unknown/No predicted functional change
**KIFC1**	43,5	43,1	53,2	41,6	alternate-3p	8	Unknown/No predicted functional change
**KMO**	10,2	N/S	11,9	N/S	alternate-3p	27	Unknown/No predicted functional change
**LRRC16B**	31,4	35,5	N/S	35,7	exon-skipping	947	Change of cellular localisation and loss of a part of CARMIL CTERM domain
**MARVELD2**	N/S	-15,1	N/S	N/S	exon-skipping	-12	Unknown/No predicted functional change
**MSS51**	-10,3	N/S	N/S	N/S	alternate-3p	-221	Loss of a signal peptide, loss of a MYND finger domain
**MT1G**	-21,7	N/S	N/S	-13,8	alternate-3p	-1	Unknown/No predicted functional change
**MXD3**	39,5	34,2	42,7	35,3	intron-retention	195	Unknown/No predicted functional change
**NFKB1**	15,0	N/S	N/S	N/S	alternate-3p	1	Unknown/No predicted functional change
**NOMO2**	N/S	N/S	21,1	N/S	exon-skipping	167	Gain of a signal peptide
**PANX2**	10,1	N/S	N/S	N/S	exon-skipping	-134	Gain of innexin domain and of 2 transmembrane domains

**PHF14**	-12,1	-14,0	N/S	N/S	exon-skipping	-285	Unknown/No predicted functional change
**PRPF4B**	-34,5	-31,5	N/S	-27,0	alternate-5p+intron-retention	No protein to a 1007 AA protein	Gain of a tyrosine kinase protein (long isoform: NMD)
**PTPN1**	-22,0	N/S	N/S	N/S	exon-skipping	-73	Loss of a part of Y-phosphatase domain
**RAB7A**	-17,5	N/S	N/S	N/S	alternate-5p	-47	Loss of half the Ras domain
**RARB**	-12,1	N/S	N/S	N/S	alternate-5p	-112	Loss of a part of ZF-C4 domain (binding to DNA)
**RINT1**	-11,7	N/S	N/S	N/S	alternate-5p+alternate-3p+multiple-exon-skipping	-425	Loss of NLS and loss of a part of RINT1 TIP1 domain
**SCRIB**	-18,3	-16,8	N/S	-17,9	exon-skipping	-25	Unknown/No predicted functional change
**SFMBT1**	-10,1	N/S	N/S	N/S	exon-skipping	-96	Loss of SAM1 domain
**SHQ1**	-13,5	-14,2	N/S	-13,1	alternate-3p	89	Unknown/No predicted functional change
**SLIT2**	62,9	N/S	N/S	43,6	exon-skipping	8	Unknown/No predicted functional change
**SPAG4**	-27,2	N/S	-33,6	N/S	alternate-5p	-77	Unknown/No predicted functional change
**SPINT2**	N/S	-11,8	N/S	N/S	exon-skipping	-57	Loss of Kunitz domain (inhibition of proteases)
**SUZ12**	-12,7	N/S	N/S	N/S	exon-skipping	-23	Unknown/No predicted functional change
**TPSB2**	N/S	N/S	19,3	N/S	alternate-3p	152	Gain of signal peptide, gain of complete trypsin domain
**VPS13A**	-21,2	-16,2	-24,6	-18,0	exon-skipping	-39	Unknown/No predicted functional change
**WASF2**	-28,6	-14,5	N/S	-13,6	exon-skipping	-217	Loss of the WH2 motif
**ZGRF1**	15,5	17,4	N/S	14,3	exon-skipping	No protein to a 2104 AA protein	Gain of a protein with 2 AAA domains, a GRF zinc finger motif and a DUF2439 domain (short isoform: NMD)
**ZNF35**	12,3	N/S	N/S	N/S	exon-skipping	160	Cytoplasmic protein (short isoform) goes to non-cytoplasmic (long isoform)
**ZNF445**	-19,8	N/S	N/S	-20,2	alternate-5p	-12	Unknown/No predicted functional change
**ZNF451**	-15,6	N/S	N/S	N/S	exon-skipping	-48	Unknown/No predicted functional change
**ZNF532**	-14,5	-12,7	N/S	N/S	alternate-5p	-910	Unknown/No predicted functional change
**ZNF552**	-10,6	N/S	-16,4	-12,3	alternate-5p	4	Unknown/No predicted functional change

Supplementary table 3 – List of tumor suppressors for which AS is dysregulated in various types of HCC

THBV/NNoV			THCV/NNoV		THBV&HCV/NNoV	TNoV/NNoV		
ABI2	EZH1	RAB7A	AFAP1L2	SAMD9L	CASP8	ABI2	HPGD	SRPX
ACHE	EZH2	RAP1GAP	BARD1	SCRIB	CTNND1	AFAP1L2	IKZF1	STRADA
AFAP1L2	FAS	RARB	BIN1	SEPT4	FHIT	AGTR1	IKZF2	TBRG1
AGTR1	FLNA	RASSF1	BRCA1	SHQ1	HACE1	ALDH1A2	ING1	TCF3
ALPL	FOXP1	RBL2	CD44	SIRT3	ITGA7	ALPL	ING4	TCF7L2
ARID2	GADD45A	RBM6	CDKN2A	SMARCB1	KRIT1	ARNTL	IRF3	TFPI2
ARNTL	GLS2	RBMX	CEACAM1	SPINT2	LIMA1	BARD1	ITGA7	THRB
AXIN1	GPC3	RINT1	CHEK1	TBRG1	NDRG2	BCL2L11	KDM8	TRIT1
BARD1	GSN	RNF111	CIZ1	TCF3	NF2	BIN1	KMT2C	TSC1
BCL2L11	GSTT1	RNH1	COPS2	TCF7L2	TET2	BLNK	KRIT1	VDR
BIN1	GTPBP4	RPL10	CREM	TP73		BRCA1	LRP1B	VEGFA
BLNK	HPGD	RUNX3	CSMD1	TRIT1		BRF1	MAD1L1	VEZT
BRCA1	HTATIP2	SAFB	CTNNA3	TSC2		CADM2	MFSD2A	VPS53
BRF1	ING1	SAMD9L	DIABLO	VDR		CASP8	MLH1	WWOX
BTK	ING3	SCRIB	DLG1	VEGFA		CCDC136	MT1G	XAF1
CABLES1	ING4	SDHA	DNMT3B	VEZT		CCNDBP1	MTSS1	ZMYND11
CADM2	IRF3	SEMA3B	DOK1	WNK2		CD4	MTUS1	ZNF382
CASP8	ITGA7	SEPT4	EMP1			CD44	NDRG2	
CCAR1	ITGB1	SHQ1	ESR1			CDK2	NF2	
CCNDBP1	KANK1	SIRT2	FLNA			CEACAM1	NPAS2	
CD44	KDM8	SIRT3	GLS2			CHEK1	NRCAM	
CDK2	KLF6	SKP2	GPC3			CIZ1	NUMB	
CEACAM1	KMT2C	SLIT2	GSN			CREM	PARK2	
CHEK1	KRIT1	SMARCB1	HPGD			CSMD1	PBRM1	
CHEK2	MAD1L1	SPOP	IGFALS			CTCFL	PLAGL1	
CIZ1	MBD4	SRPX	ING1			CTNNA3	PLCB3	
CNDP2	MFSD2A	STRADA	ING3			CTNND1	PML	
CREM	MME	SUZ12	ING4			CUX1	PPARA	
CSMD1	MT1G	TBRG1	IRF3			CYLD	PRR5	
CTCF	MTSS1	TCF3	ITGA7			DICER1	PTCH1	
CTNNA3	MTUS1	TCF7L2	KANK1			DLG1	PTPN6	
CTNND1	NDRG2	TES	KDM8			DNMT3B	RAP1GAP	
CYLD	NF1	TET2	KMT2C			ECT2	RASSF1	
DDB2	NF2	TFPI2	KRIT1			EDA2R	RASSF4	
DLG1	NFKB1	THRB	MAD1L1			EGR2	RBM6	
DMD	NUMB	TRIM35	MLH1			EHF	RNH1	
DNAJA3	PANX2	TRIT1	MTSS1			EI24	RPL10	
DNMT3B	PBRM1	TSC1	NDRG2			ESR1	SCRIB	
EEF1A1	PDCD4	UHRF2	NF1			EZH1	SEC14L2	
EGR2	PHACTR4	UVRAG	NF2			EZH2	SEMA3B	
EHD3	PLAGL1	VEGFA	NUMB			FOXP1	SEPT4	
EHF	PPARA	VEZT	PBRM1			GLS2	SHQ1	
EI24	PPP2R1B	VPS53	PPARA			GPC3	SIRT3	
EMP1	PTCH1	WWOX	PTPN6			GSN	SLIT2	
EPB41	PTPN1	XAF1	RASSF4			GSTT1	SMARCA4	
ESR1	PTPN6	ZNF382	RPL10			HOPX	SMARCB1	

Supplementary table 4 - List of oncogenes for which AS is dysregulated in various types of HCC

THBV/NNoV	THCV/NNoV	THBV&HCV/NNoV	TNoV/NNoV
CBFB	ETS1	CBFB	AXL
DEK	KRAS		CBFB
ERBB2	MYH11		MDM2
ERG	TSC2		MYH11
ETS1			PML
KRAS			
MDM2			
MYH11			
TAL1			

Supplementary table 5 - List of kinases for which AS is dysregulated in various types of HCC

THBV/NNoV		THCV/NNoV	THBV&HCV/NNoV	TNoV/NNoV	
ALPK1	MAST4	AURKA	AURKB	AURKA	MPP1
AURKA	MELK	AURKB	CDK1	AURKB	MPP3
AURKB	MORN4	CAMK2D	PI4KAP1	BRSK2	MPP4
AURKC	MPP3	CDC42BPA	PIK3CB	CAMK2D	MVK
BRSK2	MVK	CDK1	RPS6KA4	CAMK2G	NEK2
BTK	NRBP2	CDK10	SLAMF7	CD244	NRBP2
CAMK2D	PANK1	CHKB		CDC42BPA	PAPSS2
CD244	PAPSS2	DGKZ		CDK1	PASK
CD84	PASK	DGUOK		CDK10	PI4KAP1
CDC42BPA	PFKM	DMPK		CDK2	PIK3CG
CDK1	PI4KAP1	FES		CDK4	PRPF4B
CDK10	PIK3CB	FYN		CDKL3	PRPS2
CDK14	PIP4K2C	GK5		CERKL	PTK7
CDK2	PIP5K1A	IP6K2		CHKB	RIPK2
CDK20	PRKDC	KHK		CLK3	SGK3
CDK4	PRPF4B	MAGIX		CLK4	SLAMF7
CDKL3	PRPS1	MAP3K6		CSNK1G1	SPHK2
CHKB	PRPS2	MORN1		DGKG	SRPK1
CIT	RIPK1	MORN4		DGKH	STK16
CMPK1	RIPK2	PAPSS2		DGKZ	TK2
DGKG	SGK1	PI4KAP1		DGUOK	TNK2
DGKZ	SGK3	PIK3CB		DSTYK	ULK3
DGUOK	SLAMF6	PRPF4B		FASTK	VRK2
EIF2AK2	SLAMF7	PTK6		FES	WNK4
FES	SPHK2	RIPK1		FGGY	
FYN	SRPK1	RPS6KA4		FYN	
GK5	STK16	SPHK2		GK5	
GNE	TANC1	STK16		IKBKB	
HCK	TESK2	ULK3		IP6K2	
IKBKB	TK2	WNK1		KHK	
IKBKE	TNK2	WNK2		KSR1	
IP6K2	ULK3	WNK4		MAP3K6	
KHK	VRK2			MAPK13	
KSR1	WNK1			MARK1	
MAGIX	WNK3			MAST4	
MAPK13	WNK4			MELK	
MARK3				MORN4	

Supplementary table 6 - List of transcription factors for which AS is dysregulated in various types of HCC

THBV/NNoV				THCV/NNoV		THBV&HCV/NNoV	TNoV/NNoV		
ARHGAP5	KBTBD4	RNH1	ZNF35	BCL11A	TCEA3	ARHGAP5	ARNTL	NCOR1	ZBTB24
ARNTL	KDM4A	RRN3	ZNF382	CARD8	TCF20	HDGFRP2	ATXN3	NFAT5	ZBTB38
ARNTL2	KDM5D	RUNX3	ZNF408	CCNL2	TCF3	KAT6A	BCL11A	NFATC4	ZBTB41
BCL11A	KRBOX4	SFMBT1	ZNF419	CNOT1	TFDP2	LIMA1	BRF1	NFE2	ZFP64
BRF1	LMO2	SMARCE1	ZNF445	CNOT8	TP73	MXD3	C3orf67	NFE2L2	ZMYM6
BTBD1	LRRC16B	SOX13	ZNF451	CREM	UBAC2	NLRC5	CARD8	NFIA	ZMYND11
C3orf67	LRRFIP2	SPOP	ZNF502	CRIP2	UTY	SMARCC2	CBFA2T2	NFYA	ZNF107
CAND1	MKL1	SREBF1	ZNF512	E2F7	VDR	SUPT20H	CCNL2	NFYC	ZNF138
CARD8	MLXIPL	STAT6	ZNF532	ETS1	XPNPEP3	TLE2	CDIP1	NLRC5	ZNF169
CBFA2T2	MORF4L2	SUPT20H	ZNF550	FHL2	YY1AP1	ZBTB24	CNOT8	NLRP6	ZNF195
CCNL2	MPND	TAF11	ZNF552	FOSB	ZBTB38	ZMYM1	CREB3L4	NPAS2	ZNF200
CDIP1	MSS51	TAF6	ZNF567	FOSL1	ZEB1	ZMYND8	CREM	NR1I3	ZNF233
CIITA	MXD3	TAL1	ZNF618	FOXM1	ZHX3	ZNF211	CRIP2	NR2C1	ZNF248
CLOCK	NCOA3	TBX3	ZNF707	GATA3	ZNF138	ZNF552	CTCFL	NR3C1	ZNF266
CNOT1	NCOR1	TCEA3	ZNF793	HDGFRP2	ZNF169	ZNF667	CUX1	PDLIM2	ZNF274
CNOT8	NFAT5	TCF20	ZNF85	HMGB2	ZNF195		E2F6	PDLIM5	ZNF285
CREM	NFATC3	TCF3	ZNF852	HNF1B	ZNF248		E2F7	PHC2	ZNF331
CRIP2	NFIA	TEAD2	ZSCAN2	HOXB6	ZNF532		EHF	PLAG1	ZNF346
CTCF	NFKB1	TFDP2		ING1	ZNF550		ELK1	PLAGL1	ZNF382
E2F6	NFYA	TFEB		ING4	ZNF559		FHL2	POGZ	ZNF408
E2F7	NLRC5	TFEC		IRF3	ZNF707		FOSB	PPARA	ZNF431
EHF	NLRP6	THRB		JMJD1C			FOSL1	PQBP1	ZNF445
ELF1	NR1D1	TLE2		KDM5D			FOXM1	PRDM16	ZNF446
ELK1	NR1I3	TOX4		LRRC16B			FOXP1	RARG	ZNF502
ERG	NR2C1	TRIM35		LRRFIP2			FOXP2	REST	ZNF512
ETS1	NR3C1	UBAC2		MARVELD2			GATA3	RNH1	ZNF527
FHL2	NR3C2	USF2		MLXIPL			HDGFRP2	RRN3	ZNF550
FOSB	PDDC1	UTY		MORF4L2			HMGB2	SMARCE1	ZNF552
FOSL1	PDLIM2	WIZ		MXD3			HOXB6	STAT6	ZNF582
FOXK2	PDLIM5	XPNPEP3		MYNN			IKZF1	SUPT20H	ZNF618
FOXM1	PEPD	YY1AP1		NCOA1			IKZF2	TAF6	ZNF707
FOXP1	PHC2	ZBTB24		NCOA3			ING1	TCEA3	ZNF738
GABPA	PHF14	ZBTB38		NCOR1			ING4	TCF12	ZNF79
GATA3	PLAG1	ZBTB41		NFYA			IRF3	TCF20	ZNF793
GCFC2	PLAGL1	ZEB1		NR1I3			IVNS1ABP	TCF3	ZNF821
GNE	POGZ	ZEB2		NR2C1			JMJD1C	TEAD2	ZSCAN2
HDGFRP2	PPARA	ZFP64		NR3C1			KDM5D	TEAD4	
HMGB2	PPARD	ZHX3		PHC2			LMO2	TFDP2	
HNF1B	PQBP1	ZNF107		PHF14			LRRC16B	THRB	
HOXB6	PRDM16	ZNF131		PLAG1			LRRFIP2	TLE2	
ING1	PRRC2B	ZNF195		POGZ			MKL1	UBAC2	
ING4	RARA	ZNF200		PPARA			MLXIPL	UTY	
IRF3	RARB	ZNF211		PQBP1			MORF4L2	VDR	
IRF6	RARG	ZNF233		PRRC2B			MPND	XPNPEP3	
IVNS1ABP	RBL2	ZNF248		PRRC2C			MXD3	YY1AP1	
JMJD1C	RELA	ZNF331		RRN3			NCOA3	ZBTB17	

Supplementary table 7 - List of genes for which AS is dysregulated in all types of HCC

ASEs misregulated in the four types of HCC				
ACP1	CENPL	INCENP	OGFOD2	SLC2A6
ACSL4	CEP164	ISYNA1	OSBPL6	SLC7A7
ASPM	CLN3	ITGA7	PARPBP	SORT1
ATXN2L	DHRS4L2	KIAA0895L	PCYT2	ST3GAL3
AURKB	ENO3	KIAA1324	PDE4DIP	STAU2
C1orf86	FAM86C1	KIFC1	PHF3	STK25
C5orf42	FDFT1	KRIT1	PI4KAP1	TAMM41
C6orf201	FETUB	MDK	PIK3R3	TJP2
CARD14	FN1	MICU1	PMM2	VEGFB
CARKD	GOLGA2	MSTO1	POLL	VPS13A
CCDC64	GPR116	MXD3	PORCN	WASH2P
CD27-AS1	HDGFRP2	NDRG2	RCC1	WASH7P
CDC45	HNRNPUL1	NEB	RGS3	ZFYVE19
CDK1	IL17RE	NF2	SEC31B	ZNF655
CDS2	IL32	NIPA2	SKA2	ZNF692

Supplementary table 8 - List of genes uniquely dysregulated in HBV-associated HCC

ASEs misregulated in only THBV/NNoV										
A2M	BAZ2A	CR2	F7	GUSBP4	LOC100506054	NKTR	POFUT2	RSU1	STEAP1B	TSPO
AAK1	BBS4	CRYZ	F9	HADHA	LOC155060	NMRAL1	POGK	RTN2	STOM	TTC7A
ABCB7	BCL2L14	CSDE1	FADS6	HAL	LOC619207	NOL11	POLB	RTN3	STRAP	TUFT1
ABCD3	BCS1L	CSF3R	FAM153A	HAUS6	LOC654433	NOVA1	POLDIP3	RUNX3	STT3A	TXN2
ABHD10	BDP1	CTCF	FAM179B	HBEGF	LRIF1	NPEPPS	POLR2M	SAFB	STX16	UACA
ABI3BP	BIVM	CTH	FAM198A	HCK	LRRC61	NPHP3	POLR3D	SAP130	STX5	UBAP1
ACAD8	BRAP	CTNNAL1	FAM199X	HEATR5A	LSS	NR1D1	PPARD	SBF1	SULF1	UBAP2L
ACADSB	BRWD3	CWF19L2	FAM206A	HEATR5B	LTBP3	NR3C2	PPIL2	SBNO1	SUMF2	UBE2K
ACCS	BSDC1	CXorf38	FAM210A	HECTD1	LUZP6	NRBF2	PPM1E	SCAND2P	SUN1	UBL7
ACHE	BTBD1	CYBRD1	FAM228A	HECTD3	LYPLA1	NRG3	PPP1R10	SCARB1	SUN2	UBQLN4
ACLY	BTK	CYFIP2	FAM3C	HERC4	LYSMD3	NSG1	PPP1R8	SDC4	SURF4	UCHL5
ACMSD	C10orf118	CYP2B6	FAM57A	HHAT	MAGT1	NUCB2	PPP2R1A	SDHA	SUV420H1	UHRF2
ACSS2	C14orf159	CYP2C8	FAM63B	HINT3	MAN2B2	NUDT9	PPP2R1B	SDHAP2	SUZ12	USF2
ACTA2	C15orf39	CYP39A1	FAM65C	HMGCS2	MANEAL	NUP50	PPP2R5D	SEC23A	SWAP70	USP15
ACTL6A	C16orf62	CYP4F2	FAS	HNRNPC	MAP4	NUP54	PPP4R1	SEC23IP	SYNE1	USP36
ACVRL1	C21orf58	DAO	FASTKD2	HNRNPH3	MAPKAP1	OGFOD1	PRG4	SEC24D	SYNJ2	UVRAG
ADAM10	C2CD5	DAXX	FBLN7	HP1BP3	MARCH6	OGT	PRKCSH	SEC61A1	SYP	VAC14
ADAR	C6orf106	DAZAP2	FBXL5	HSD11B1L	MARK3	ORC4	PRKDC	SECISBP2	TAF11	VAMP7
ADCY3	CABLES1	DCAF7	FBXO3	HSD17B4	MATN3	OXR1	PRMT2	SEMA5B	TAL1	VASH2
ADCY5	CABYR	DCDC5	FGFR4	HSD3B7	MAVS	P2RX4	PRMT5	SENP6	TANC1	VMP1
ADH6	CACNA1H	DDB2	FKBP10	HSDL2	MBD1	P4HA2	PRNP	SEPT10	TARS	VNN2
ADPGK	CALCOCO2	DEK	FKBP5	HSF4	MBD4	PADI1	PRPS1	SERPINF2	TBC1D4	VPS16
AGPAT1	CALD1	DET1	FKRP	HSPH1	MBNL3	PAK4	PRSS8	SETD4	TBRG4	VPS26A
AGXT2L1	CAND1	DGAT2	FLJ00385	HTATIP2	MCCC1	PANK1	PSMC1	SFMBT1	TBX3	VPS41
ALB	CANX	DGCR2	FLYWCH1	HYLS1	MCCC2	PANX2	PSMD3	SFXN4	TCP11L1	VSIG4
ALDH18A1	CAP1	DHCR24	FLYWCH2	IAH1	MCOLN3	PARP9	PSME4	SGCD	TCTN3	VWF
ALDH1A3	CAPN1	DHX36	FNBP1L	IARS	MEAF6	PCBP1-AS1	PTCD3	SGCE	TDRD9	WAC
ALDH2	CBWD3	DHX40	FOLH1	IBTK	MEF2D	PCGF3	PTDSS1	SGK1	TECPR2	WDR44
ALPK1	CCAR1	DHX58	FOPNL	ICAM1	MEMO1	PCGF6	PTPLAD1	SGSM3	TES	WDR6
AMD1	CCDC111	DIAPH1	FOXK2	IDH3A	METTL23	PCK1	PTPN1	SH3PXD2A	TESK2	WIPF2
AMDHD1	CCDC135	DIO1	FTSJ2	IDI1	METTL5	PCK2	PTPN21	SHANK2	TFEB	WIZ
AMPD3	CCDC25	DMD	FUT6	IFNLR1	MFN1	PCNX	PVRL4	SIGLEC10	TFEC	WNK3
ANKHD1	CCDC90B	DMGDH	FZD6	IGSF9	MFSD12	PCNXL4	PWWP2A	SIMC1	TGM2	WSB2
ANKIB1	CCM2	DNAJA1	G3BP1	IKBKE	MGAT4B	PCYOX1	PYGL	SIRT2	THEMIS	XIRP1
ANKRD19P	CCNG1	DNAJA3	G6PC	IL10RA	MGME1	PDCD1	RAB18	SKIV2L2	THSD7B	XPC
ANKS6	CCSER2	DNAJC19	G6PC3	IL18RAP	MINK1	PDCD4	RAB2B	SKP2	THTPA	XRCC6
ANXA8	CD200	DNM2	GABPA	IL6ST	MIOS	PDDC1	RAB5A	SLAMF6	TIMM10B	XRN2
AOX1	CD300A	DNPEP	GABPB1	INTS9	MLIP	PDE3B	RAB5B	SLC13A5	TLR2	YWHAE
AP1AR	CD34	DPP3	GADD45A	IREB2	MLLT4	PDE8B	RAB6A	SLC15A4	TLR4	ZBTB21

AP5M1	CD68	DRAM2	GCFC2	IRF6	MLPH	PDGFC	RAB7A	SLC17A1	TM9SF2	ZC3H14
APH1A	CD84	DSCR3	GDA	ITCH	MME	PDHB	RABEP1	SLC23A2	TMBIM1	ZC3HC1
API5	CDC25B	DUS3L	GDE1	ITFG3	MMP19	PDHX	RABGAP1	SLC25A13	TMEM116	ZCCHC6
APMAP	CDK14	DUSP16	GDI2	ITGB1	MMRN2	PDXDC1	RAI2	SLC25A43	TMEM136	ZCWPW1
APP	CDK20	DYM	GEM	IWS1	MNS1	PDXDC2P	RAP1B	SLC27A2	TMEM161B	ZDHHC19
AQP3	CDS1	DYNC1LI1	GEMIN7	JKAMP	MOB2	PDZK1	RARA	SLC2A14	TMEM167B	ZDHHC23
AQP6	CECR2	DYNC2LI1	GEMIN8	KBTBD4	MON1B	PEPD	RARB	SLC2A2	TMEM170A	ZDHHC5
ARCN1	CELF2	EBF4	GFM2	KCNT2	MRC2	PFKFB4	RARS	SLC30A5	TMEM216	ZEB2
ARFGAP3	CERS2	ECD	GFOD2	KDM4A	MROH7	PFKM	RASSF6	SLC35B2	TMEM254	ZFYVE9
ARHGEF10L	CFHR4	EEF1A1	GLE1	KIAA0125	MRPL3	PGGT1B	RBBP5	SLC38A2	TMEM254-AS1	ZMAT5
ARID2	CHD6	EFTUD2	GLYATL1	KIAA0232	MSS51	PGRMC1	RBL2	SLC38A7	TMEM30A	ZNF131
ARL5A	CHEK2	EHD3	GMPS	KIAA1598	MST1P2	PHACTR4	RBM23	SLC44A3	TMEM44	ZNF302
ARL6IP1	CHRD	EIF2AK2	GNAS	KIDINS220	MTA1	PI16	RBM24	SLC7A9	TMEM63B	ZNF326
ARL8B	CIDEC	ELAC2	GNB1	KLF6	MTF2	PIBF1	RBM42	SLC9A8	TMEM66	ZNF35
ARNTL2	CIITA	ELF1	GNB5	KLF7	MTFR1	PIGA	RBM47	SLMAP	TMEM8A	ZNF410
ASB13	CIRH1A	ELN	GNE	KLHL12	MTM1	PIGG	RBMX	SMARCD1	TMPRSS4	ZNF415
ASH2L	CIT	EML4	GNL3	KLHL2	MTMR14	PIGT	RCBTB2	SMEK1	TMTC1	ZNF419
ASPH	CLASRP	ENOPH1	GNS	KLRD1	MYD88	PIK3R5	RDH16	SMIM8	TNKS1BP1	ZNF451
ASPN	CLOCK	ENOX2	GOLGA5	KPNB1	MYLIP	PIP4K2C	RELA	SNAP23	TOLLIP	ZNF567
ASUN	CMPK1	ENPP2	GOLPH3L	KRBOX4	MYO9B	PIP5K1A	RETSAT	SNTA1	TOP2B	ZNF839
ATG16L1	CNDP2	EOGT	GOT2	LAMP1	NAA60	PLA2G1B	RGPD1	SOX13	TOR3A	ZNF85
ATP2B2	CNPY3	EPB41	GPNMB	LASP1	NAE1	PLEKHH3	RHPN2	SPAG9	TOX4	ZNF852
ATP2B4	CNTRL	ERBB2	GPR137	LGMN	NARG2	PLEKHO2	RINT1	SPECC1L	TPRG1L	
ATP2C1	COG3	ERBB3	GPR161	LILRB1	NARS	PLS3	RMDN2	SPOP	TRAP1	
ATP5SL	COL18A1	ERC1	GPR180	LILRB2	NBEAL1	PMPCA	RNF111	SPSB3	TRAPPC11	
ATP6V1C1	COL1A2	ERG	GRAMD1B	LILRB3	NCAPG2	PMS2CL	RNF4	SRD5A1	TRAPPC6A	
ATP7B	COL6A6	ERMP1	GRB2	LILRB5	NDC1	PMS2P5	RNGTT	SREBF1	TRAPPC6B	
ATP9B	COQ5	ERVK13-1	GSS	LIPC	NDUFS5	PNPO	RPH3AL	SRP54	TRIM35	
AURKC	CORO1C	ESYT2	GSTO2	LIPG	NET1	PODN	RRAS2	SRP72	TRIM41	
AXIN1	CPD	F11R	GTPBP4	LMAN2L	NFATC3	PODXL	RRM1	SSFA2	TRIM5	
BACE1	CPSF7	F3	GUCY1B3	LMBR1	NFKB1	POFUT1	RSAD1	ST6GAL1	TRPV3	

Supplementary table 9 - List of genes uniquely dysregulated in HCV-associated HCC

ASEs misregulated in only THCV/NNoV

AASS	FCGR3A	MORN1	SLC10A7
ANXA2	FGFR1OP	MRPL55	SMARCAD1
ATP6V0B	FLJ39739	MTHFSD	SNX5
CACNB4	FUT10	MYNN	SPINT2
CCDC7	GLT1D1	N4BP2L1	SUSD1
CD22	HNRNPH1	NAALAD2	TFPI
CDKN2A	IGFALS	NCOA1	TGIF1
CHRNA4	IL9R	NEIL2	TP73
COPS2	INIP	PHGDH	TROVE2
DIABLO	JHDM1D	PHYHD1	TSC2
DMPK	KAZN	PPP3CB	WNK2
DNAJC18	KLHDC1	PRRC2C	WNT2
DOK1	LINC00875	PTK6	ZNF106
EFCAB1	LOC388152	R3HDM2	ZNF30
EMR1	MAEL	RACGAP1	ZNF559
FAM83A-AS1	MARS	RYK	ZNF568
FBXO28	MARVELD2	SAMD4A	ZNF638

Supplementary table 10 - List of genes uniquely dysregulated in HBV&HCV-associated HCC

ASEs misregulated in only THBV&HCV/NNoV		
ALAD	HACE1	RGN
ALOX12P2	KAT6A	RPL15
ARHGAP44	KIFC3	SMARCC2
ATP6V1H	KMT2E	SNRPN
C7orf63	LANCL1	SYBU
CD28	LIMA1	TCIRG1
CD8B	LIN54	TPSB2
CNTNAP3	MCTP1	TRABD2A
CYP21A2	MIB2	VANGL1
DHX35	NBPF10	WDPCP
FAM135A	NBPF8	WEE2-AS1
FAM13A	NCEH1	XRCC1
FHIT	NOMO2	ZCCHC7
FYB	NR2F1-AS1	ZFAT
GDPD5	ORAI2	ZMYM1
GIGYF2	PACSIN3	ZMYND8
GNL1	PMEPA1	ZNF182
GRB10	RBMS1	ZNF667

Supplementary table 11 - List of genes uniquely dysregulated in virus-free HCC

ASEs misregulated in only TNoV/NNoV				
AADAT	CSNK1G1	JOSD2	PIK3CG	STAC3
ACSF2	CTCFL	KANSL1	PLCB3	STAG3L3
ACSL6	CUL9	KCNK17	PLEKHS1	STARD3
ADORA2A	CUTC	KCNMB3	PML	SUV39H2
AKAP17A	CUX1	KIAA0391	POLR1B	SVEP1
ALDH1A2	CWF19L1	KIAA0895	PPCS	TBC1D17
AMZ2	CYB561D1	KIF20B	PPP2R2B	TBCK
APOBEC3B	DCAF11	KLHL3	PRDX1	TCF12
ARHGAP30	DCUN1D2	KLRF1	PROM1	TDP1
ARMC7	DDX11L1	LGALS9	PRR5	TEAD4
ATP13A4	DGKH	LIMCH1	PSMD13	TESPA1
ATRNL1	DICER1	LINC00910	PSPH	TIMD4
ATXN1	DIO2	LOC100287534	PSTPIP1	TM4SF19
ATXN3	DIO3AS	LOC400927	PTK7	TM7SF2
ATXN7L3	DIP2A	LOXL2	PTPN20A	TMEM67
AXL	DMAP1	LOXL3	PTPN20B	TNFRSF25
BBS1	DSTYK	LRP1B	PTPRS	TOMM40L
BBX	ECT2	LRRC23	PYCR1	TPK1
BCAS1	EDA2R	LRRC7	RAB37	TREML1
BCL7B	EPHA5	LRRK1	RABL5	TRIM39
BDKRB2	ERCC6L2	LRRN2	RAD51AP1	TRPC4
BPY2	FAM213B	LUZP2	RAPGEF1	TTLL3
BTBD7	FAM21A	MARK1	RAPGEF3	TTLL7
BTNL8	FAM227A	MAZ	RBM14-RBM4	TTYH1
BTRC	FAM92A1	MCM2	REPS1	UBE2J2
C12orf65	FASTK	MDH1B	RERG	UBE2W
C1orf109	FBXL2	MICAL2	REST	UNK
C1QTNF6	FGGY	MPC2	RGL4	UPF3B
C21orf91	FOXP2	MPP1	RGS11	USP25
CADPS	FRMD1	MPP4	RIMBP2	UXS1
CALML4	FUZ	MRPL52	RINL	VIPR2
CAMK2G	GALNT14	MS4A14	RPGRIP1	VWA1
CAPN12	GALNT6	MSANTD2	RPL23AP7	VWDE
CCDC136	GAPVD1	MTCH1	RRAGC	WASF3
CCDC149	GATS	NDUFAF7	RTEL1-TNFRSF6B	WASH1
CCDC91	GDAP1	NECAB2	RUNDC3B	WDR20
CD320	GEMIN5	NEK2	SCAMP4	WDR81
CD4	GEN1	NFASC	SCARA5	WSCD1
CDAN1	GLIS3	NFATC4	SEC14L2	ZBTB17

CDC25A	GPM6A	NFE2	SENP7	ZDHHC15
CEP97	GRAP2	NFE2L2	SH2D1A	ZMYM6
CERKL	GUCY1B2	NFYC	SIGMAR1	ZMYND11
CFLAR	HDAC7	NHSL2	SLC12A4	ZNF266
CHN1	HDAC8	NLGN4X	SLC14A1	ZNF274
CHRNA7	HELB	NPAS2	SLC25A12	ZNF285
CIB2	HERC2P3	NRCAM	SLC25A19	ZNF334
CLCN7	HFE	NXF3	SLC26A3	ZNF346
CLEC4A	HLA-G	ORAOV1	SLC30A10	ZNF431
CLIP1	HMCN1	P2RX2	SLC35F6	ZNF446
CLK3	HNRNPA1	P4HA1	SLC35G1	ZNF454
CLK4	HOPX	PAK3	SLFN12	ZNF527
CNIH2	HPSE	PAOX	SMARCA4	ZNF534
CNTN5	ICA1	PAPD5	SNAP91	ZNF582
COL24A1	IGFLR1	PARK2	SNCAIP	ZNF738
CPA5	IKZF1	PCDH18	SNRPA1	ZNF79
CRCP	IKZF2	PDCD1LG2	SPATA9	ZNF791
CREB3L4	IL34	PDE10A	SPDL1	ZNF821
CRYZL1	INF2	PGBD2	SPEF2	ZNF823
CSF2RA	ISCU	PGPEP1	SSPO	ZSCAN25
CSF2RB	IYD	PIFO	ST7L	

SUPPLEMENTARY FIGURES

Figure S1. Characterization of splicing mysregulation in HCC. (A) Distribution of Delta PSI values in various types of HCC. **(B)** Distribution of multiple alternative splicing events (ASEs) in each gene for various types of HCC. **(C)** The splicing events lists for various types of HCC were filtered to keep only data with at least two replicates for both virus-induced HCC and virus-free healthy tissues (NNoV). Fold changes were then calculated between HCC and healthy tissues. Q-values (false-discovery rate) were calculated in order to take into account multiple statistical hypothesis testing, and results under 0.05 were considered significant. Events with a P-value of less than 0.05 were conserved. Events with expression levels higher than one TPM in at least one dataset were conserved.

Fig. S2. Characterization of ASEs that are modified in HBV- and HCVassociated HCC. (A) Gene ontology analysis for alternative splicing modifications in HBV- and HCV-associated HCC. The asterisk (*) indicates the other categories: Cellular process (GO: 0009987), cellular component organization or biogenesis (GO: 0071840), localization (GO: 0051179), locomotion (GO: 0040011) and multicellular organismal process (GO: 0032501). **(B)** Interaction network for cellular genes for which AS is modified in both HBV- and HCC-associated HCC. The network was determined by uploading the genes into STRING.

Figure S3. AS modifications in transcripts encoded by kinases and transcriptions factores in HBV- and HCV-associated HCC. (A) Alterations in the AS patterns of transcripts encoded by kinases in HBV- and HCV-associated HCC. Some transcripts have multiple ASEs that are modified. Red bars indicate negative Delta PSI values, and blue bars represent positive Delta PSI values. **(B)** Alterations in the AS patterns of transcripts encoded by transcription factors in HBV- and HCV-associated HCC.

Figure S4. Global profiling of ASE modifications in both HBV&HCVassociated HCC and virus-free-associated HCC. (A) Heatmap representation of ASEs (PSI values) for

cellular transcripts in HBV&HCV-associated HCC. Data obtained from THBV&HCV (HBV&HCV-associated HCC) tissues are shown in purple and data obtained from heathy tissues (NNoV) are shown in green. **(B)** Heatmap representation of ASEs (PSI values) for cellular transcripts in virus-free HCC. Data obtained from virus-free-associated HCC tissues are shown in yellow and data obtained from heathy tissues (NNoV) are shown in green.

Figure S5. RNA splicing factors in HCC. Iris Graph representing the expression profile of splicing factors for **(A)** HCV-, **(B)** HBV&HCV-, and **(C)** virus-free HCC. Differences in gene expression levels are shown on a logarithmic color scale (Log2), from red (negative changes in expression) to blue (increase in gene expression). The expression of proteins involved in splicing modulated by more than 2-fold is indicated by an asterisk.

Fig. S6. Modifications to AS of 96 transcripts in response to knockdown of splicing factors with specific siRNAs. Using specific siRNAs, twelve splicing factors (listed on the right) were individually knocked-down in different cell lines to assess their implication in splicing of 96 differents transcripts (listed at the top). Individual knockdowns and ASEs are shown to indicate which knockdowns caused a shift in alternative splicing in various cell lines (PC-3, SKOV3, NIH:OVCAR-3, MDA-MB-231, MCF7). Each column represents a distinct knockdown performed with specific siRNAs. The changes in PSI values are displayed. The map represents the changes in PSI values in a color-coded scale. White areas indicate no shifts. Asterisks indicate transcripts for which AS was altered in HCV-associated HCC.

A

∣ΔPSI∣	THBV/NNoV	THCV/NNoV	THBV&HCV/NNoV	TNoV/NNoV
[10-20[	2190	844	61	1638
[20-30[	699	335	77	520
[30-40[	207	117	41	170
[40-50[	75	38	22	51
[50-60[	45	23	8	33
[60-70[	18	10	4	11
[70-80[	14	11	4	10
[80-90[	2	2	3	5
[90-100]	0	1	0	0

B

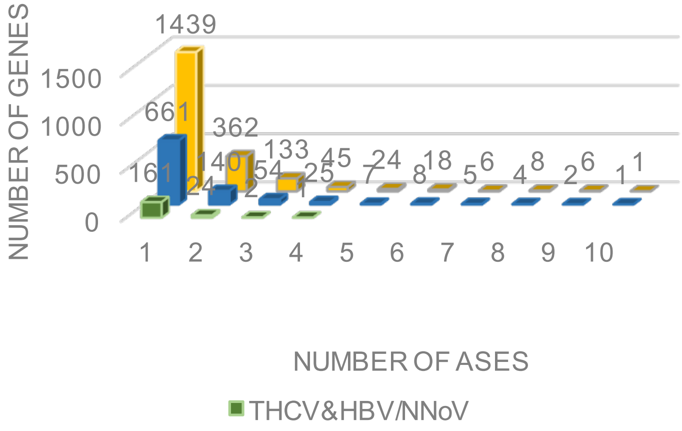

C

	THBV NNoV	THCV NNoV	THBV&HCV NNoV	TNoV NNoV
Total ASEs	42 659	45 362	37 389	42 826
n ≥ 2 in both conditions	39 971	39 582	31 404	40 198
p ≤ 0,05	9 129	6 325	3 028	8 676
q ≤ 0,05	6 381	2 818	318	6 071
TPM ≥ 1 in at least one condition	6 379	2 815	315	6 070
Delta PSI ≤ -10 or ≥ 10 Alternative splicing events	3 250	1 381	220	2 438
Genes	2 051	907	188	1 517

Fig. S1

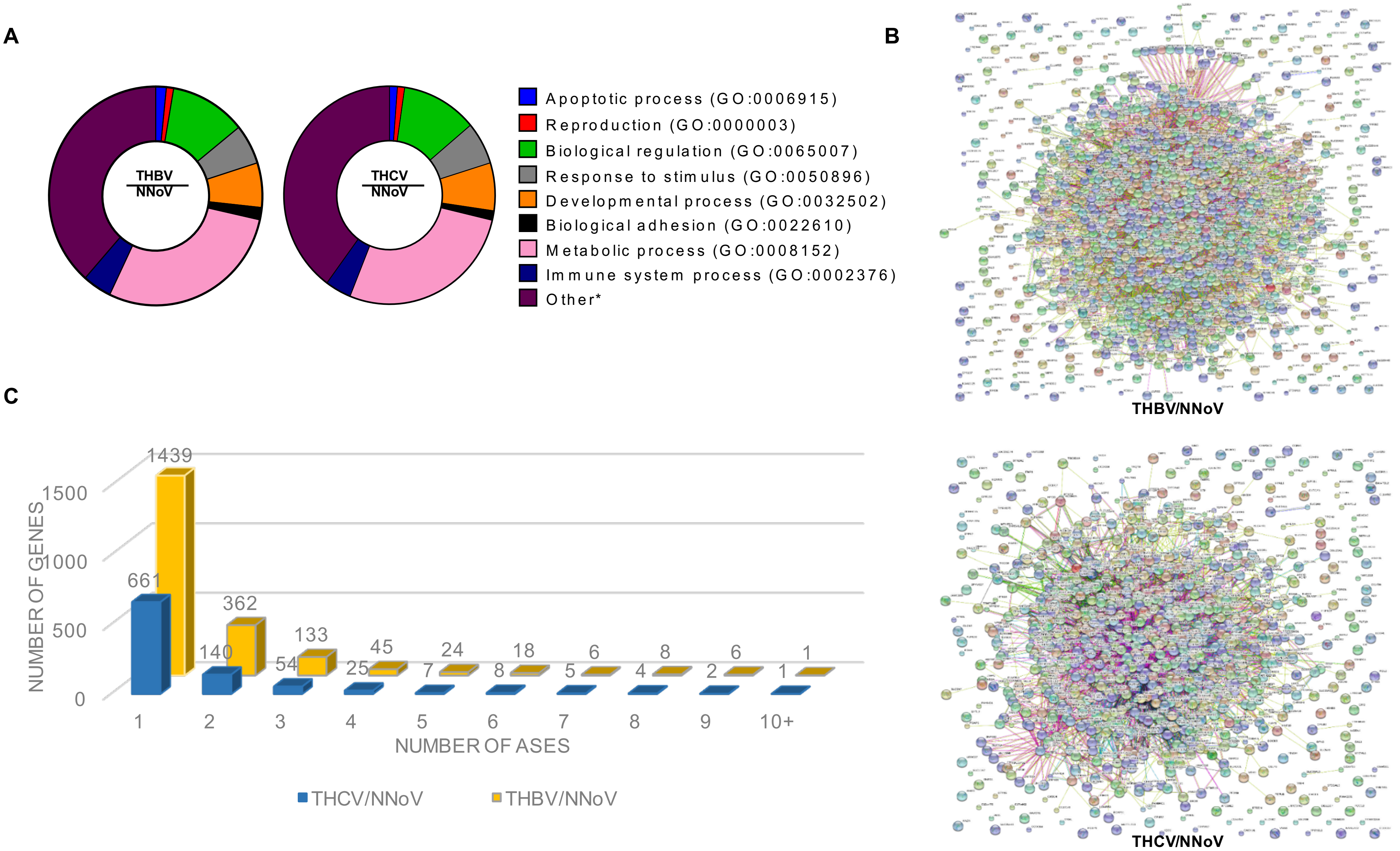
A
THBV
NNoV
THCV
NNoV
Apoptotic process (GO:0006915)
Reproduction (GO:0000003)
Biological regulation (GO:0065007)
Response to stimulus (GO:0050896)
Developmental process (GO:0032502)
Biological adhesion (GO:0022610)
Metabolic process (GO:0008152)
Immune system process (GO:0002376)
Other*
B
THBV/NNoV
THCV/NNoV
C
NUMBER OF GENES
0
500
1000
1500
1439
661
362
140
133
54
45
25
24
7
18
8
6
5
8
4
6
2
1
1
1 2 3 4 5 6 7 8 9 10+
NUMBER OF ASES
THCV/NNoV
THBV/NNoV

Fig. S2

A

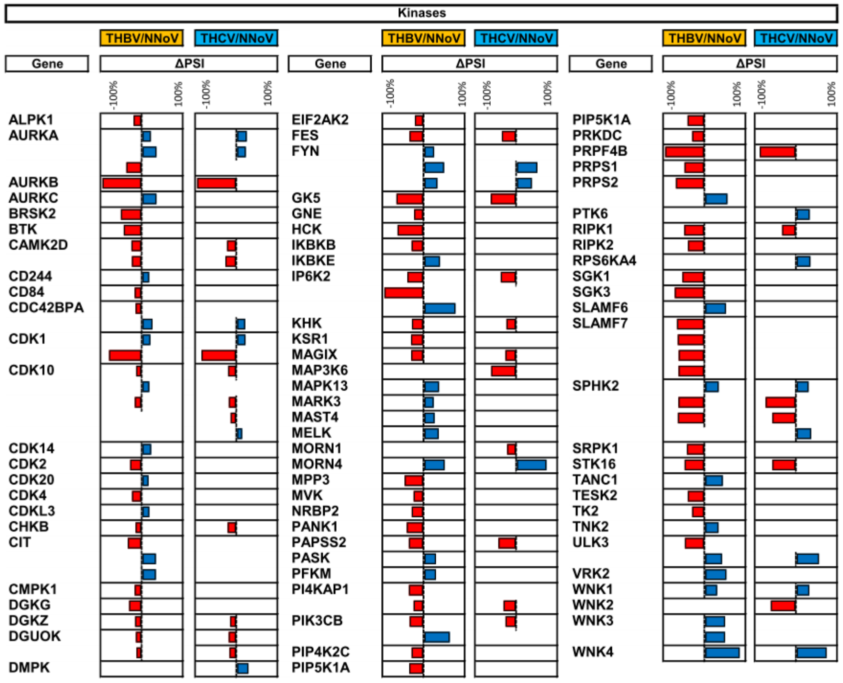

Kinases
THBV/NNoV
THCV/NNoV
Gene
ΔPSI
-100%
100%
ALPK1
AURKA
AURKB
AURKC
BRSK2
BTK
CAMK2D
CD244
CD84
CDC42BPA
CDK1
CDK10
CDK14
CDK2
CDK20
CDK4
CDKL3
CHKB
CIT
CMPK1
DGKG
DGKZ
DGUOK
DMPK
EIF2AK2
FES
FYN
GK5
GNE
HCK
IKBKB
IKBKE
IP6K2
KHK
KSR1
MAGIX
MAP3K6
MAPK13
MARK3
MAST4
MELK
MORN1
MORN4
MPP3
MVK
NRBP2
PANK1
PAPSS2
PASK
PFKM
PI4KAP1
PIK3CB
PIP4K2C
PIP5K1A
PIP5K1A
PRKDC
PRPF4B
PRPS1
PRPS2
PTK6
RIPK1
RIPK2
RPS6KA4
SGK1
SGK3
SLAMF6
SLAMF7
SPHK2
SRPK1
STK16
TANC1
TESK2
TK2
TNK2
ULK3
VRK2
WNK1
WNK2
WNK3
WNK4

B

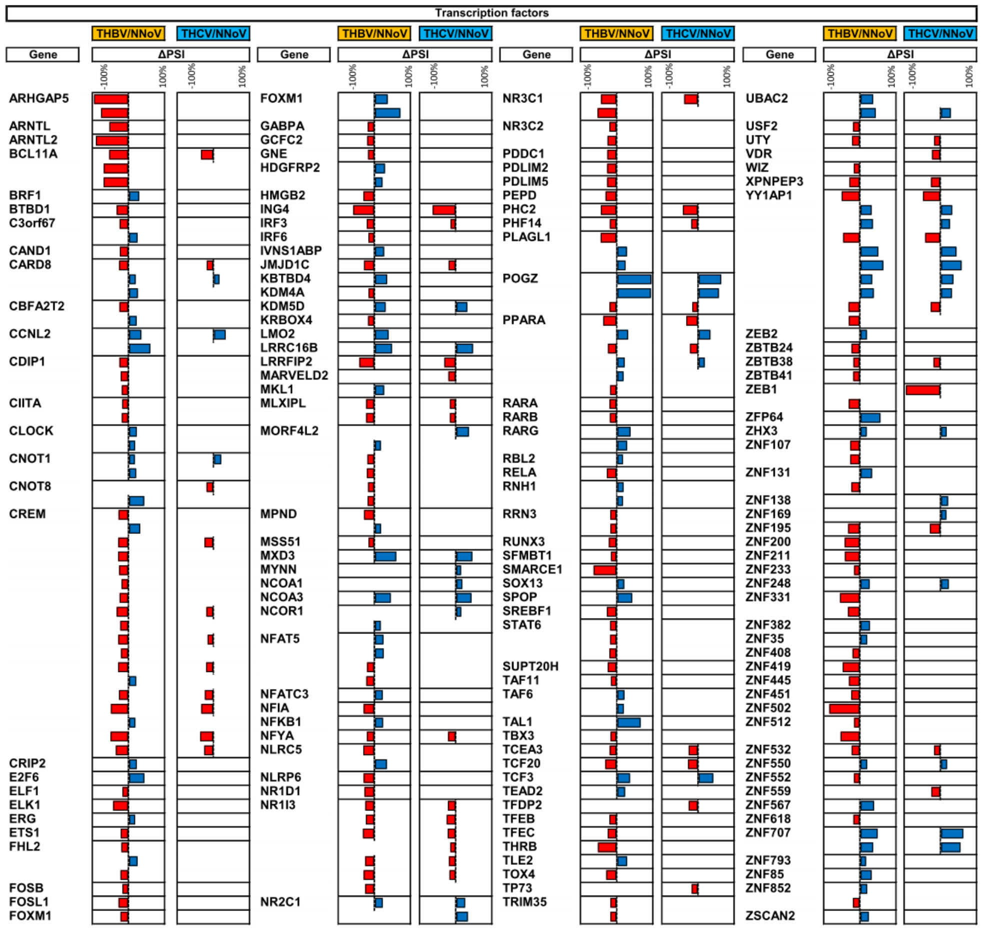

Transcription factors
THBV/NNoV
THCV/NNoV
Gene
ΔPSI
-100%
100%
ARHGAP5
ARNTL
ARNTL2
BCL11A
BRF1
BTBD1
C3orf67
CAND1
CARD8
CBFA2T2
CCNL2
CDIP1
CIITA
CLOCK
CNOT1
CNOT8
CREM
CRIP2
E2F6
ELF1
ELK1
ERG
ETS1
FHL2
FOSB
FOSL1
FOXM1
FOXM1
GABPA
GCFC2
GNE
HDGFRP2
HMGB2
ING4
IRF3
IRF6
IVNS1ABP
JMJD1C
KBTBD4
KDM4A
KDM5D
KRBOX4
LMO2
LRRC16B
LRRFIP2
MARVELD2
MKL1
MLXIPL
MORF4L2
MPND
MSS51
MXD3
MYNN
NCOA1
NCOA3
NCOR1
NFAT5
NFATC3
NFIA
NFKB1
NFYA
NLRC5
NLRP6
NR1D1
NR1I3
NR2C1
NR3C1
NR3C2
PDDC1
PDLIM2
PDLIM5
PEPD
PHC2
PHF14
PLAGL1
POGZ
PPARA
RARA
RARB
RARG
RBL2
RELA
RNH1
RRN3
RUNX3
SFMBT1
SMARCE1
SOX13
SPOP
SREBF1
STAT6
SUPT20H
TAF11
TAF6
TAL1
TBX3
TCEA3
TCF20
TCF3
TEAD2
TFDP2
TFEB
TFEC
THRB
TLE2
TOX4
TP73
TRIM35
UBAC2
USF2
UTY
VDR
WIZ
XPNPEP3
YY1AP1
ZEB2
ZBTB24
ZBTB38
ZBTB41
ZEB1
ZFP64
ZHX3
ZNF107
ZNF131
ZNF138
ZNF169
ZNF195
ZNF200
ZNF211
ZNF233
ZNF248
ZNF331
ZNF382
ZNF35
ZNF408
ZNF419
ZNF445
ZNF451
ZNF502
ZNF512
ZNF532
ZNF550
ZNF552
ZNF559
ZNF567
ZNF618
ZNF707
ZNF793
ZNF85
ZNF852
ZSCAN2

Fig. S3

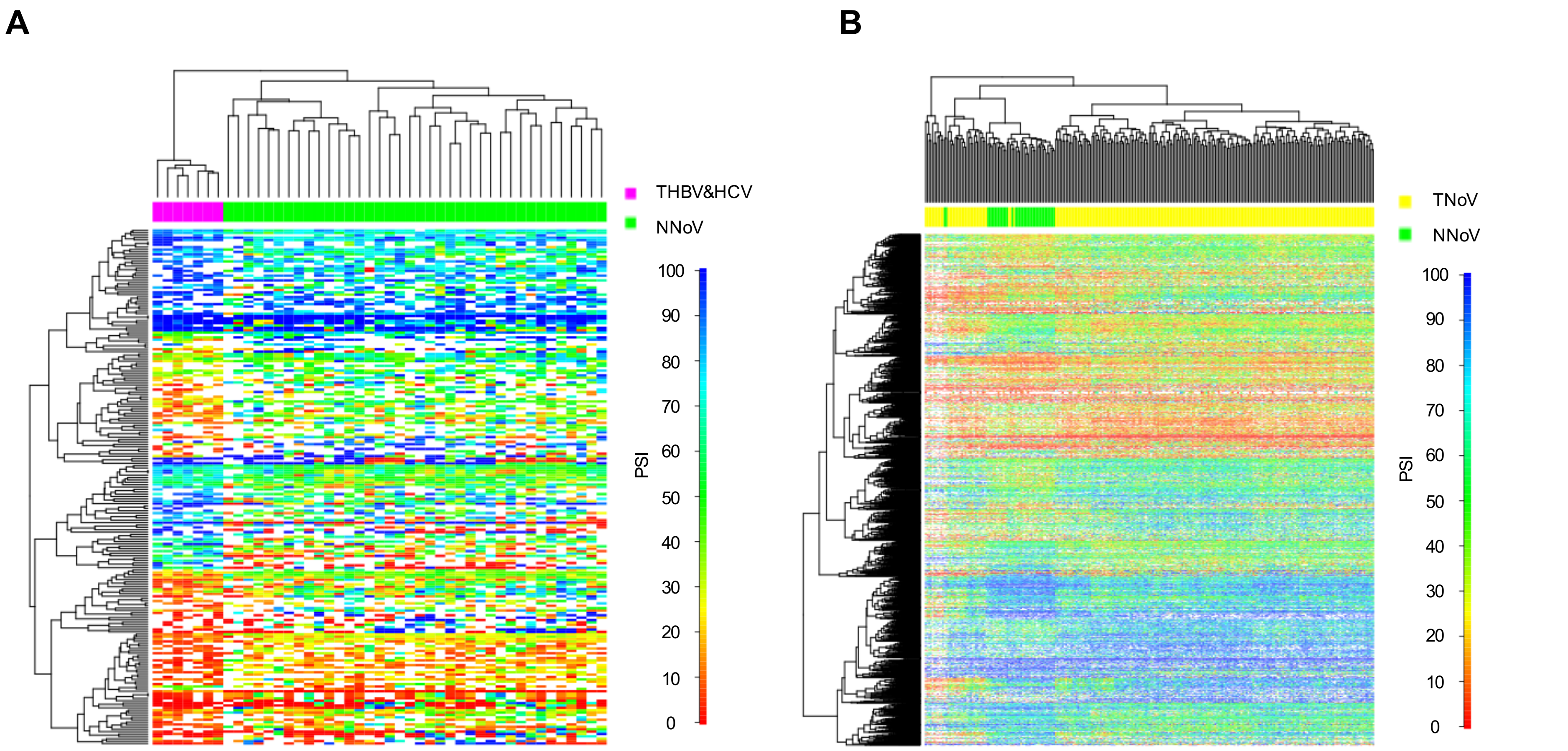
A
THBV&HCV
NNoV
PSI
100
90
80
70
60
50
40
30
20
10
0
B
TNoV
NNoV
PSI
100
90
80
70
60
50
40
30
20
10
0

Fig. S4

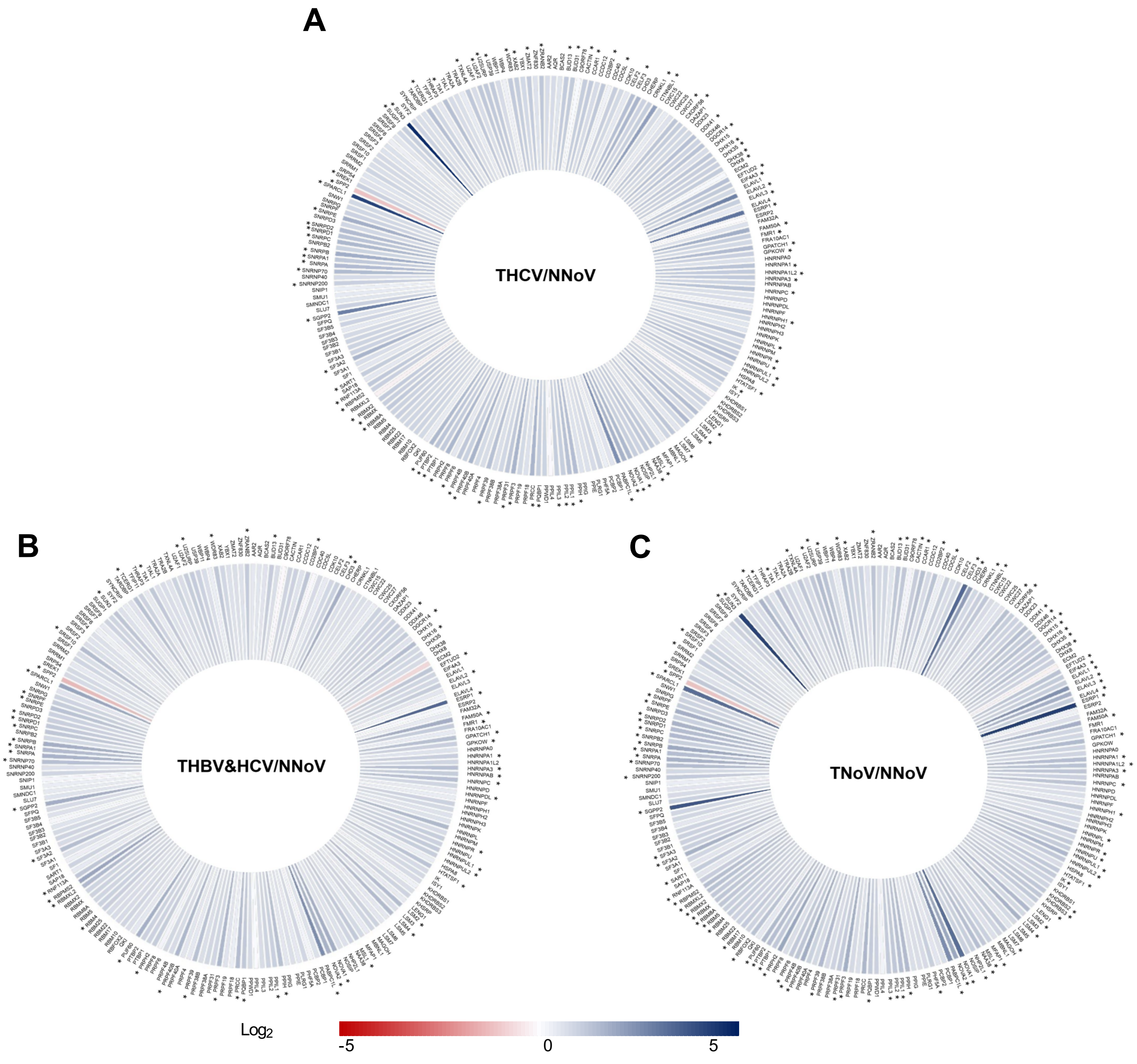

Fig. S5

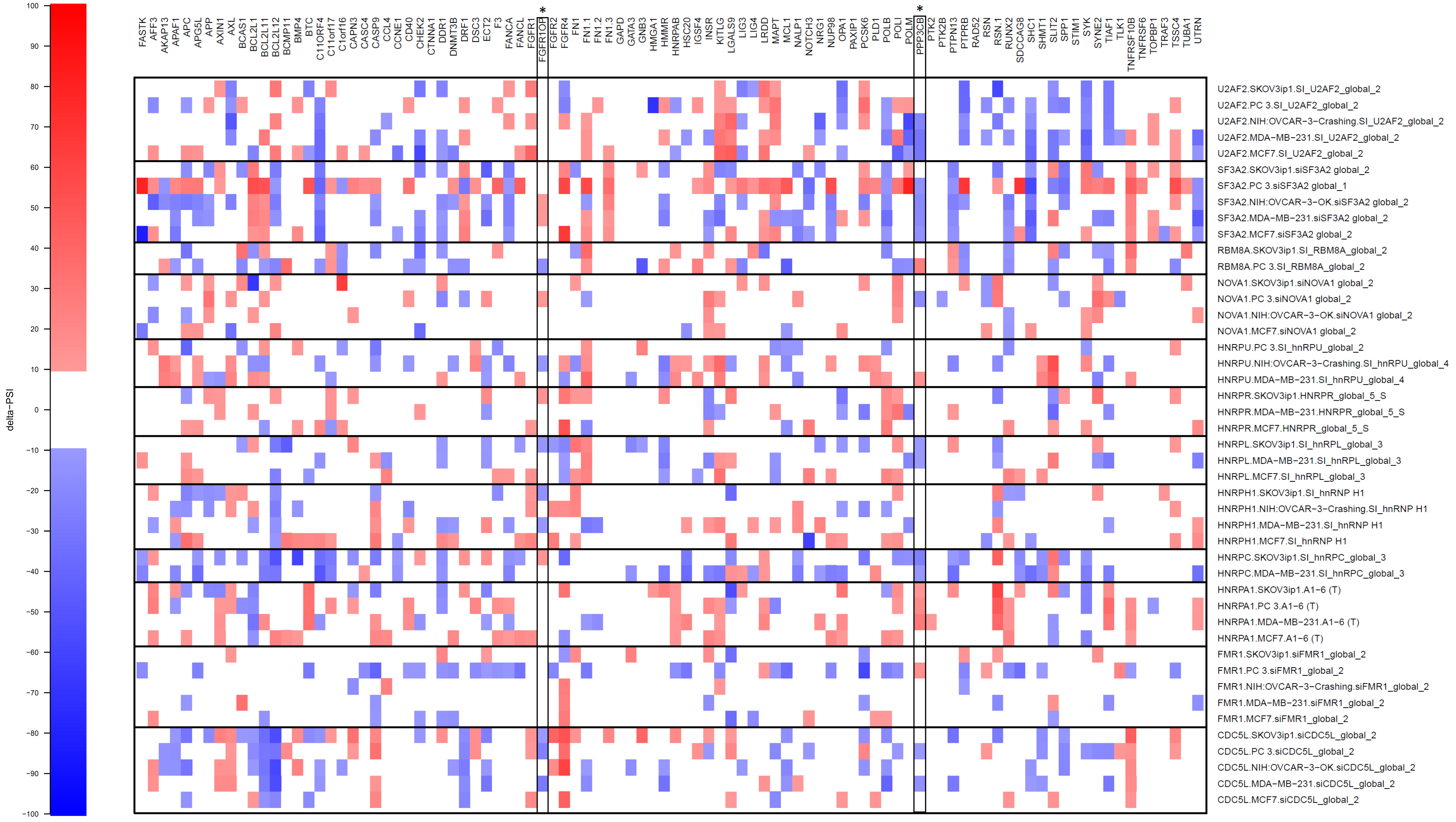

100
90
80
70
60
50
40
30
20
10
0
-10
-20
-30
-40
-50
-60
-70
-80
-90
-100
delta-PSI
*
*
FASTK
AFF3
AKAP13
APAF1
APC
APG5L
APP
AXIN1
AXL
BCAS1
BCL2L1
BCL2L11
BCL2L12
BCMP11
BMP4
BTC
C11ORF4
C11orf17
C1orf16
CAPN3
CASC4
CASP9
CCL4
CCNE1
CD40
CHEK2
CTNNA1
DDR1
DNMT3B
DRF1
DSC3
ECT2
F3
FANCA
FANCL
FGFR1
FGFR1OP
FGFR2
FGFR4
FN1
FN1.1
FN1.2
FN1.3
GAPD
GATA3
GNB3
HMGA1
HMMR
HNRPAB
HSC20
IGSF4
INSR
KITLG
LGALS9
LIG3
LIG4
LRDD
MAPT
MCL1
NALP1
NOTCH3
NRG1
NUP98
OPA1
PAXIP1
PCSK6
PLD1
POLB
POLI
POLM
PPP3CB
PTK2
PTK2B
PTPN13
PTPRB
RAD52
RSN
RSN.1
RUNX2
SDCCAG8
SHC1
SHMT1
SLIT2
SPP1
STIM1
SYK
SYNE2
TIAF1
TLK1
TNFRSF10B
TNFRSF6
TOPBP1
TRAF3
TSSC4
TUBA1
UTRN
U2AF2.SKOV3ip1.SI_U2AF2_global_2
U2AF2.PC 3.SI_U2AF2_global_2
U2AF2.NIH:OVCAR−3−Crashing.SI_U2AF2_global_2
U2AF2.MDA−MB−231.SI_U2AF2_global_2
U2AF2.MCF7.SI_U2AF2_global_2
SF3A2.SKOV3ip1.siSF3A2 global_2
SF3A2.PC 3.siSF3A2 global_1
SF3A2.NIH:OVCAR−3−OK.siSF3A2 global_2
SF3A2.MDA−MB−231.siSF3A2 global_2
SF3A2.MCF7.siSF3A2 global_2
RBM8A.SKOV3ip1.SI_RBM8A_global_2
RBM8A.PC 3.SI_RBM8A_global_2
NOVA1.SKOV3ip1.siNOVA1 global_2
NOVA1.PC 3.siNOVA1 global_2
NOVA1.NIH:OVCAR−3−OK.siNOVA1 global_2
NOVA1.MCF7.siNOVA1 global_2
HNRPU.PC 3.SI_hnRPU_global_2
HNRPU.NIH:OVCAR−3−Crashing.SI_hnRPU_global_4
HNRPU.MDA−MB−231.SI_hnRPU_global_4
HNRPR.SKOV3ip1.HNRPR_global_5_S
HNRPR.MDA−MB−231.HNRPR_global_5_S
HNRPR.MCF7.HNRPR_global_5_S
HNRPL.SKOV3ip1.SI_hnRPL_global_3
HNRPL.MDA−MB−231.SI_hnRPL_global_3
HNRPL.MCF7.SI_hnRPL_global_3
HNRPH1.SKOV3ip1.SI_hnRNP H1
HNRPH1.NIH:OVCAR−3−Crashing.SI_hnRNP H1
HNRPH1.MDA−MB−231.SI_hnRNP H1
HNRPH1.MCF7.SI_hnRNP H1
HNRPC.SKOV3ip1.SI_hnRPC_global_3
HNRPC.MDA−MB−231.SI_hnRPC_global_3
HNRPA1.SKOV3ip1.A1−6 (T)
HNRPA1.PC 3.A1−6 (T)
HNRPA1.MDA−MB−231.A1−6 (T)
HNRPA1.MCF7.A1−6 (T)
FMR1.SKOV3ip1.siFMR1_global_2
FMR1.PC 3.siFMR1_global_2
FMR1.NIH:OVCAR−3−Crashing.siFMR1_global_2
FMR1.MDA−MB−231.siFMR1_global_2
FMR1.MCF7.siFMR1_global_2
CDC5L.SKOV3ip1.siCDC5L_global_2
CDC5L.PC 3.siCDC5L_global_2
CDC5L.NIH:OVCAR−3−OK.siCDC5L_global_2
CDC5L.MDA−MB−231.siCDC5L_global_2
CDC5L.MCF7.siCDC5L_global_2

Fig. S6
